# Dual TTK/PLK1 inhibition has potent anticancer activity in TNBC as monotherapy and in combination

**DOI:** 10.3389/fonc.2024.1447807

**Published:** 2024-08-09

**Authors:** Elisa Zanini, Nicole Forster-Gross, Felix Bachmann, Adrian Brüngger, Paul McSheehy, Karine Litherland, Karin Burger, Anna C. Groner, Mila Roceri, Luc Bury, Martin Stieger, Nicole Willemsen-Seegers, Jos de Man, Diep Vu-Pham, Helma W. E. van Riel, Guido J. R. Zaman, Rogier C. Buijsman, Laurenz Kellenberger, Heidi A. Lane

**Affiliations:** ^1^ Basilea Pharmaceutica International Ltd, Allschwil, Switzerland; ^2^ Oncolines B.V., Oss, Netherlands; ^3^ Crossfire Oncology B.V., Oss, Netherlands

**Keywords:** BAL0891, mitotic checkpoint inhibitor, PLK1, SAC, TTK, Mps-1

## Abstract

**Background:**

Threonine tyrosine kinase (TTK) and polo-like kinase 1 (PLK1) are common essential kinases that collaborate in activating the spindle assembly checkpoint (SAC) at the kinetochore, ensuring appropriate chromosome alignment and segregation prior to mitotic exit. Targeting of either TTK or PLK1 has been clinically evaluated in cancer patients; however, dual inhibitors have not yet been pursued. Here we present the *in vitro* and *in vivo* characterization of a first in class, dual TTK/PLK1 inhibitor (BAL0891).

**Methods:**

Mechanism of action studies utilized biochemical kinase and proteomics-based target-engagement assays. Cellular end-point assays included immunoblot- and flow cytometry-based cell cycle analyses and SAC integrity evaluation using immunoprecipitation and immunofluorescence approaches. Anticancer activity was assessed *in vitro* using cell growth assays and efficacy was evaluated, alone and in combination with paclitaxel and carboplatin, using mouse models of triple negative breast cancer (TNBC).

**Results:**

BAL0891 elicits a prolonged effect on TTK, with a transient activity on PLK1. This unique profile potentiates SAC disruption, forcing tumor cells to aberrantly exit mitosis with faster kinetics than observed with a TTK-specific inhibitor. Broad anti-proliferative activity was demonstrated across solid tumor cell lines *in vitro*. Moreover, intermittent intravenous single-agent BAL0891 treatment of the MDA-MB-231 mouse model of TNBC induced profound tumor regressions associated with prolonged TTK and transient PLK1 in-tumor target occupancy. Furthermore, differential tumor responses across a panel of thirteen TNBC patient-derived xenograft models indicated profound anticancer activity in a subset (~40%). Using a flexible dosing approach, pathologically confirmed cures were observed in combination with paclitaxel, whereas synergy with carboplatin was schedule dependent.

**Conclusions:**

Dual TTK/PLK1 inhibition represents a novel approach for the treatment of human cancer, including TNBC patients, with a potential for potent anticancer activity and a favorable therapeutic index. Moreover, combination approaches may provide an avenue to expand responsive patient populations.

## 1 Introduction

The Spindle Assembly Checkpoint (SAC, also known as the ‘mitotic checkpoint complex’) is a surveillance mechanism that delays mitotic progression until all chromosomes are correctly attached to spindle microtubules, in order to ensure accurate chromosome segregation during cell division (1). Aberrant inactivation of the SAC results in premature anaphase onset, and therefore mis-segregation of erroneously attached chromosomes (2). This consequently leads to chromosomal instability (CIN) and aneuploidy/polyploidy, characteristics shared by the majority of human cancers despite normal cells being highly intolerant to both phenotypes (3, 4). Overexpression and dysregulation of several mitotic regulators, including the SAC kinases Threonine Tyrosine Kinase (TTK) and Polo-Like Kinase 1 (PLK1), are mechanisms through which tumor cells can tolerate high aneuploidy and CIN (5–7). However, tumor cells do have an apparent CIN threshold, above which viability is impaired (8).

The dual-specificity protein kinase TTK, also known as monopolar spindle 1 kinase (Mps-1), is a serine/threonine/tyrosine kinase that plays a key role in the recruitment of components of the SAC complex (9–11) through phosphorylation of a key SAC activator, kinetochore scaffold 1 (KNL1) (12). PLK1 is a serine/threonine protein kinase that cooperates with TTK in SAC regulation (13, 14), an activity often masked by PLK1’s multiple roles in cell cycle progression from interphase to cytokinesis (14–16). Therefore, while inhibition of TTK activity compromises the SAC and leads to premature mitotic exit, inhibition of PLK1 generally causes a mitotic block. A number of TTK and PLK1 inhibitors have entered clinical development for the treatment of cancer, with the TTK inhibitors mainly explored in combination with taxanes and more recently with estrogen receptor (ER) antagonists (17). PLK1 inhibitors have been clinically evaluated as monotherapy, as well as in combination with a variety of chemotherapeutic drugs. However, development has been complicated by a narrow therapeutic index and dose-limiting toxicities (17–19) thought to be related to effects on multiple mitotic checkpoints (20). Integrated bioinformatic analyses have identified both TTK and PLK1 as differentially expressed key hub genes involved in triple-negative breast cancer (TNBC) tumorigenesis (21, 22). This is consistent with the known prognostic implications of elevated TTK and PLK1 expression, as well as the high levels of aneuploidy and CIN associated with this tumor type (20, 23). With a lack of targeted therapies (surgery, radiotherapy and chemotherapy are still the principal standard of care [SoC] options) TNBC is considered one of the most aggressive breast cancer sub-types, with frequent development of chemo- and radio-resistance and a need for further exploration of novel therapeutic targets (24–29).

Here we report the novel TTK inhibitor, BAL0891, which has an additional PLK1 inhibitory activity. We show that BAL0891 has prolonged activity on TTK but short-term effects on PLK1. This results in a more rapid disruption of the SAC than observed with a TTK-specific inhibitor, associated with potentiated aberrant mitotic progression and no indications of a PLK1-related mitotic block. Differential anti-proliferative effects are observed across a diverse panel of cancer cell lines, with potent single agent antitumor efficacy in a subset of TNBC patient-derived xenograft (PDX) models. Furthermore, synergistic anticancer responses are associated with the combination of BAL0891 with paclitaxel or carboplatin, which are SoC chemotherapeutic agents for the treatment of TNBC.

## 2 Materials and methods

### 2.1 Kinase biochemical assays

Binding to purified His-tagged TTK kinase domain or full-length biotinylated PLK1 was determined by surface plasmon resonance using Biacore T200 (Cytiva) as described (30, 31). Inhibition of full-length TTK enzyme activity was determined using an Immobilized Metal Assay for Phosphochemicals (IMAP^®^) assay (Molecular Devices). Inhibition of full-length PLK1 enzyme activity was determined using LANCE Ultra TR-FRET assay (Perkin Elmer) as described (30). Fluorescein polarization and time-resolved fluorescence were measured using an Envision multimode reader (Perkin Elmer, Waltham, MA, U.S.A.). IC50s were calculated using XLfit5 software (ID Business Solutions, Ltd., Surrey, U.K.). Kinase selectivity screens were performed at Reaction Biology (Freiburg, Germany; previously ProQinase GmbH) and Eurofins Pharma Discovery Services (Dundee, UK).

Target occupancy in cell lysates and tumor samples was determined using a proteomics-based target-engagement assay. See **Supplementary Information** for further details.

### 2.2 Cell culture

HT29 and THP-1 cell lines were acquired from ATCC (HTB-38, TIB-202) and were cultured in RPMI-1640, 10% (v/v) FCS, 1% (v/v) Penicillin/Streptomycin and 2 mM L-Glutamine and grown at 37°C in 5% CO**_2_**. All cell lines (see **Supplementary Table 6**) were tested regularly to exclude mycoplasma infection.

### 2.3 Compounds and antibodies

BAL0891 was supplied by NTRC Therapeutics B.V. (now Crossfire Oncology B.V). Onvansertib and CFI-402257 were purchased from MedChem Express, nocodazole from Sigma (#M1404), carboplatin from Qilu Pharmaceutical, and paclitaxel from Beijing Union Pharmaceutical Factory. Antibodies against BubR1 (#612502) and CDC27 (#610454) were purchased from BD Biosciences; CDC20 (#4823), GAPDH [14C10] (#2118), Histone H3 (#9717), phospho-TCTP (S46) (#5251) and TCTP (#5128) antibodies from Cell Signaling Technology (CST); Mad2L1 [EPR9852] (#171084), Cyclin B1 (#32053) and phospho-Histone H3 (#32107) antibodies from Abcam; phospho-TTK (T33, S37) (#44-1325G) and TTK (#35-9100) antibodies from ThermoFisher Scientific; normal mouse IgG (#Sc-2025) from Santa Cruz Biotechnology and CENP-C (#PD030) antibodies from MBL Life science.

### 2.4 Flow cytometry analysis

Cells were fixed in 70% high grade ethanol, washed in PBS, incubated in propidium iodide (PI) solution (50 μg/mL PI and 0.5 mg/mL RNAse A, Sigma-Aldrich, in PBS) for 30 min and analyzed using a BD FACSCanto II flow cytometer (BD Biosciences).

### 2.5 Immunological assays

Immunoblotting, SAC co-immunoprecipitation assays and immunofluorescence microscopy were performed using standard procedures as outlined in the **Supplementary Information**.

### 2.6 Animal studies

Animal studies were performed by Charles-River Laboratories (Wilmington, Massachusetts, USA; MDA-MB-231 xenograft experiments) or Crown Biosciences Inc, (Taicang, China; SK-OV-3 xenograft and TNBC PDX screening experiments). All studies were performed in adherence with local ethical rules.

5x10^6^ MDA-MB-231 cells were injected subcutaneously (s.c.) in the flank of NCr nude mice (n=8-15/group for efficacy experiments, n=2-3/group for tumor pharmacodynamic evaluation), 1x10^7^ SK-OV-3 cells were injected s.c. in the flank of Balb/c nude mice (n=8/group) and specific PDX tumor fragments (3x3x3 mm^3^) were inoculated s.c. in the flank of Balb/c nude mice or NOD/SCID mice (n=4-5/group for efficacy screening and tumor pharmacodynamic experiments, n=8/group for combination experiments). Mice were randomly grouped based on a mean tumor volume (TV) of 100-200 mm^3^. For dedicated pharmacodynamic studies, treatment began with TVs of ~400 mm^3^. Mice were culled when individual tumors exceeded 1500 mm^3^ or if the body-weight loss (BWL) was >15% for 3 consecutive days or reached 20%. Dosing holidays were given to allow recovery for BWL >10%. When the endpoint was reached, mice were culled and plasma and tissue samples obtained. Samples were snap frozen and stored at -80 °C. In some cases, tumors were formalin-fixed and paraffin-embedded (FFPE) following standard procedures. BAL0891 was dissolved in a mixture of ethanol: PEG400: 20 mM citric acid at 1: 1: 8, pH 5. Paclitaxel (6 mg/mL) was prepared using the clinical formulation (Cremophor-EL) and diluted in 0.9% saline just prior to use. Carboplatin was prepared in 5% glucose in pure water. Dosing and combination regimens for each specific experiment are outlined in the text.

TV (calculated as (W 2*L)/2, where W= width and L= length, in mm, of the tumor) and BW were measured at least twice per week, and the final efficacy was calculated as the Treatment/Control ratio (ΔT/C=[mean(T)-mean(T0)]/[mean(C)-mean(C0)]) on the day when one animal had to be culled due to a large tumor size. BW is presented as the % ΔBW and as the fractional-change (endpoint/treatment-start) for each group. As an assessment of synergy, the Clarke-Combination-Index (CCI) was applied (32) where a CCI of <-0.1 was considered synergistic, >+0.1 antagonistic, and in-between, additive.

### 2.7 Statistical analyses

The statistical significance of effects was determined using Prism software, either via a one-way analysis of variance with Holm-Sidak *post-hoc* for multiple comparisons or via a two-way ANOVA with Tukey *post-hoc* for multiple comparisons. If the data was not normally distributed (Brown-Forsythe test) a Log10 transformation was applied prior to the analysis or Brown-Forsythe and Welch ANOVA tests were used.

## 3 Results

### 3.1 BAL0891 has dual activity on TTK and PLK1, with prolonged TTK and transient PLK1 inhibition

**Figure 1:**
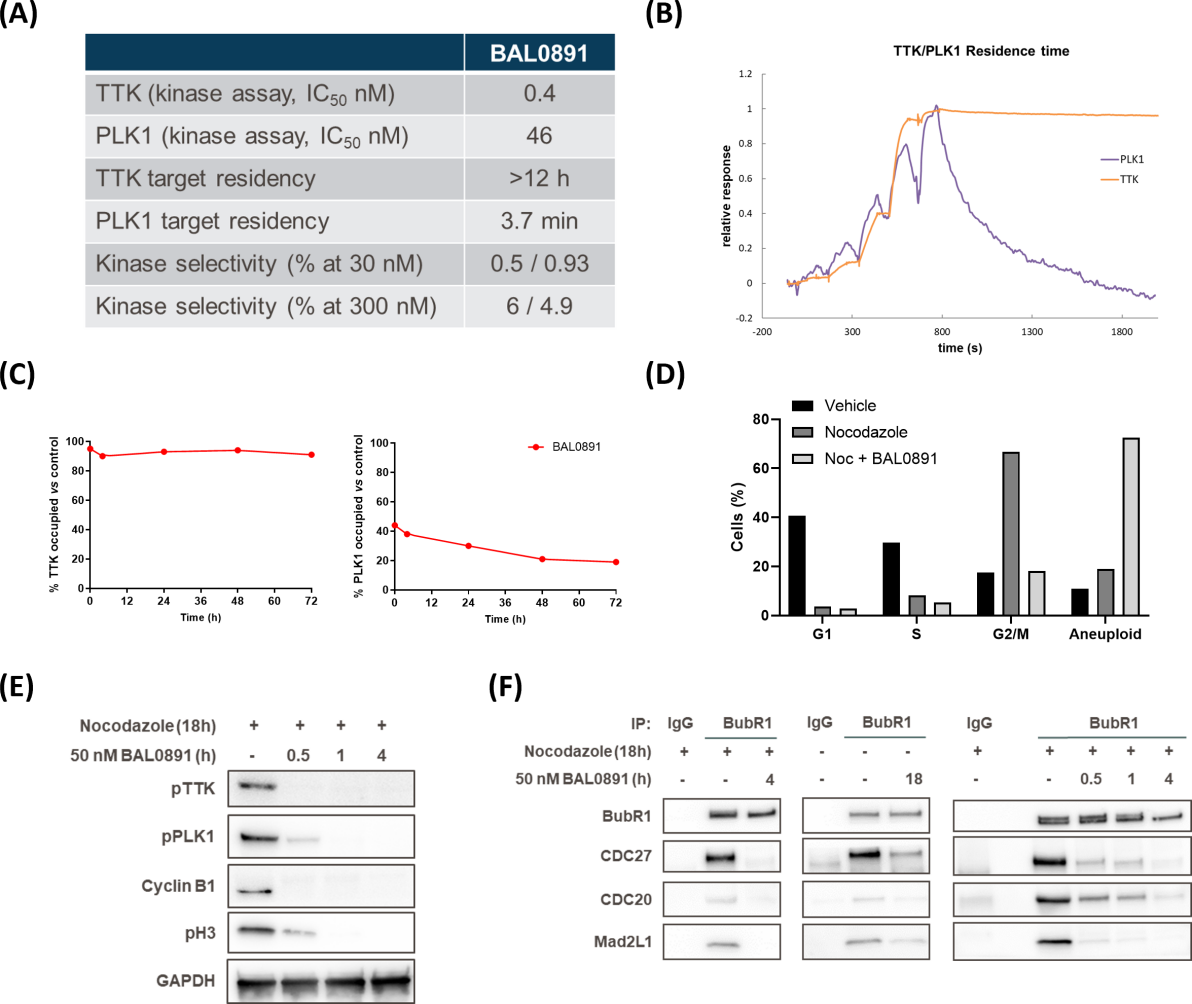
BAL0891 is a dual TTK and PLK1 inhibitor that causes rapid SAC disruption and aberrant mitotic exit. **(A)** Biochemical properties of BAL0891. **(B)** Overlay of sensorgrams of single-cycle kinetics experiments with BAL0891, using the kinase domain of TTK and full-length PLK1 on a Biacore T200. **(C)** TTK and PLK1 drug occupancy in THP-1 cells. Samples were collected 0, 4, 24, 48 and 72 h after 2 h treatment with 50 nM BAL0891 followed by wash-out. % drug-occupied TTK (left) and PLK1 (right) was calculated compared to vehicle controls. **(D)** % cells in G1, S and G2/M cell cycle phases and with higher ploidy (aneuploid) from flow cytometry profiles of HT29 cells blocked in mitosis with nocodazole (100 ng/mL, 7 h) and treated with BAL0891 (50 nM) for 18 h. **(E)** Immunoblot of mitotic markers from HT29 cell extracts blocked in mitosis with nocodazole (50 ng/mL, 18 h) and then treated with BAL0891 (50 nM) for the indicated times. GAPDH is loading control. **(F)** Co-immunoprecipitation assays using HT29 cell extracts, showing the interaction between BubR1 and the indicated SAC proteins. Cells were blocked in mitosis with nocodazole (50 ng/mL, 18 h) and treated for the indicated times with 50 nM BAL0891. An IgG antibody was used as negative control.

BAL0891 is a novel, pyrimido-indolizine, small molecule kinase inhibitor developed using structure-guided TTK target residence time optimization (33). Biochemical analyses (**Figures 1A, B**) indicated prolonged TTK target residency (>12 hours) in a surface plasmon resonance (SPR) binding assay, using a Biacore T200 and purified TTK kinase domain, with an equilibrium dissociation activity (KD) of 12.5 pM. In enzyme activity assays with purified full-length TTK, BAL0891 exhibited a half-maximum inhibitory concentration (IC50) of 0.4 nM. Interestingly, activity on purified full-length PLK1 was also observed (IC50 46 nM) associated with a reduced target residency time of 3.7 minutes and a KD of 12 nM. Activity on TTK and PLK1 was confirmed using two independent kinase assay providers, with evaluation of kinase selectivity against large kinase panels (>400 kinases) revealing low cross-reactivity (**Figure 1A**; **Supplementary Table 1**). Specifically, based on kinases exhibiting ≥50% inhibition, treatment with 30 nM BAL0891 provided a selectivity score <1%, while 300 nM BAL0891 was associated with a selectivity score of 5-6%, indicating promising selectivity. This would also be expected *in vivo*, as mouse efficacy studies indicated initial free plasma BAL0891 concentrations at efficacious doses (12.5 mg/kg IV) in the range of 60-90 nM. Of note, exposure equivalent to 300 nM cannot be achieved with tolerated doses of BAL0891 (see efficacy data).

Based on the biochemical profiling data, drug wash-out experiments in THP-1 cells were performed to test the hypothesis that BAL0891 has a potent and prolonged activity on TTK, associated with a more transient effect on PLK1, also in a cellular system. TTK and PLK1 target occupancy evaluation, using a proteomics-based assay, allowed quantification of isolated BAL0891-unoccupied kinase. Using this approach, after incubation with 50 nM BAL0891 for 2 hours, TTK was found to be almost 100% drug-occupied for a period of 4-72 hours. In contrast, PLK1 was at most <50% occupied, with evidence of recovery between 24 and 72 hours (**Figure 1C**). Hence, the cellular data appeared consistent with the biochemical profile.

### 3.2 BAL0891 induces SAC disruption and aberrant mitotic exit

The mechanism of action of BAL0891 was further evaluated using HT29 tumor cells as a model system. Treatment with the microtubule-targeting agent nocodazole caused a large (67%) accumulation in G2/M of the cell cycle, indicative of an expected stringent mitotic block (**Figure 1D**). This was associated with potent activation of TTK and PLK1, as judged by immunoblotting for phospho-TTK (Thr33, Ser37) and phospho-PLK1 (Thr210) (**Figure 1E**) which was inhibited after 4 hours BAL0891 treatment in a concentration-dependent manner (**Supplementary Figure 1A**). Interestingly, the disappearance of the mitotic markers cyclin B1 and phospho-histone H3 (pH3) was associated with maximal inhibition of both TTK and PLK1 consistent with mitotic exit (**Figure 1E**; **Supplementary Figure 1A**).

In order to evaluate the effects of BAL0891 on SAC integrity, BubR1 co-immunoprecipitation experiments were performed using HT29 tumor cells blocked in mitosis by nocodazole (**Figure 1F**, left panel) or growing asynchronously (**Figure 1F**, middle panel). In both scenarios, selected SAC components (CDC27, CDC20 and Mad2L1) were found associated with BubR1, and this was reduced following 4 hours treatment with a supra-optimal 50 nM BAL0891 concentration (see **Supplementary Figure 1A**). SAC disruption occurred in a time-dependent manner, with strong effects already observed after 30 minutes treatment (**Figure 1F**, right panel) concordant with mitotic exit as defined by reduced mitotic marker expression (**Figure 1E**). Interestingly, loss of a slower migrating CDC27 band indicative of dephosphorylation (**Supplementary Figure 1B**) was consistent with SAC disruption (34, 35).

Based on the above observations, it was expected that BAL0891-treated tumor cells would exit mitosis. Indeed, flow cytometry analyses indicated that the G2/M accumulation associated with nocodazole treatment was abrogated after 18 hours of BAL0891 treatment (**Figure 1D**) with an associated increase in aneuploid cells indicating aberrant mitotic progression and inappropriate chromosomal segregation (**Figure 1D**; **Supplementary Figure 1C**).

### 3.3 Rapid SAC disruption is associated with dual activity on TTK and PLK1

As PLK1 cooperates with TTK in SAC regulation (14), the question arose of whether dual activity on TTK and PLK1 may contribute to the potent activity of BAL0891 on the SAC. In order to address this, the specific TTK inhibitor CFI-402257, previously shown to inactivate the SAC and induce mitotic exit (36), and the PLK1-specific compound onvansertib, previously shown to induce a mitotic block (37), were used as comparators. Consistent with the literature and the many roles of PLK1 in the initiation, progression and completion of mitosis, treatment of HT29 tumor cells with onvansertib led to stabilization of mitotic markers (**Supplementary Figure 1D**) associated with an increased G2/M population by flow cytometry analysis (**Supplementary Figure 1F**). Moreover, the mitotic block induced by onvansertib was bypassed by BAL0891 treatment (**Supplementary Figures 1E, F**) indicating that, although BAL0891 does have PLK1 activity, its activity on TTK is dominant, presumably due to a higher potency and more prolonged TTK target occupancy (see above).

**Figure 2:**
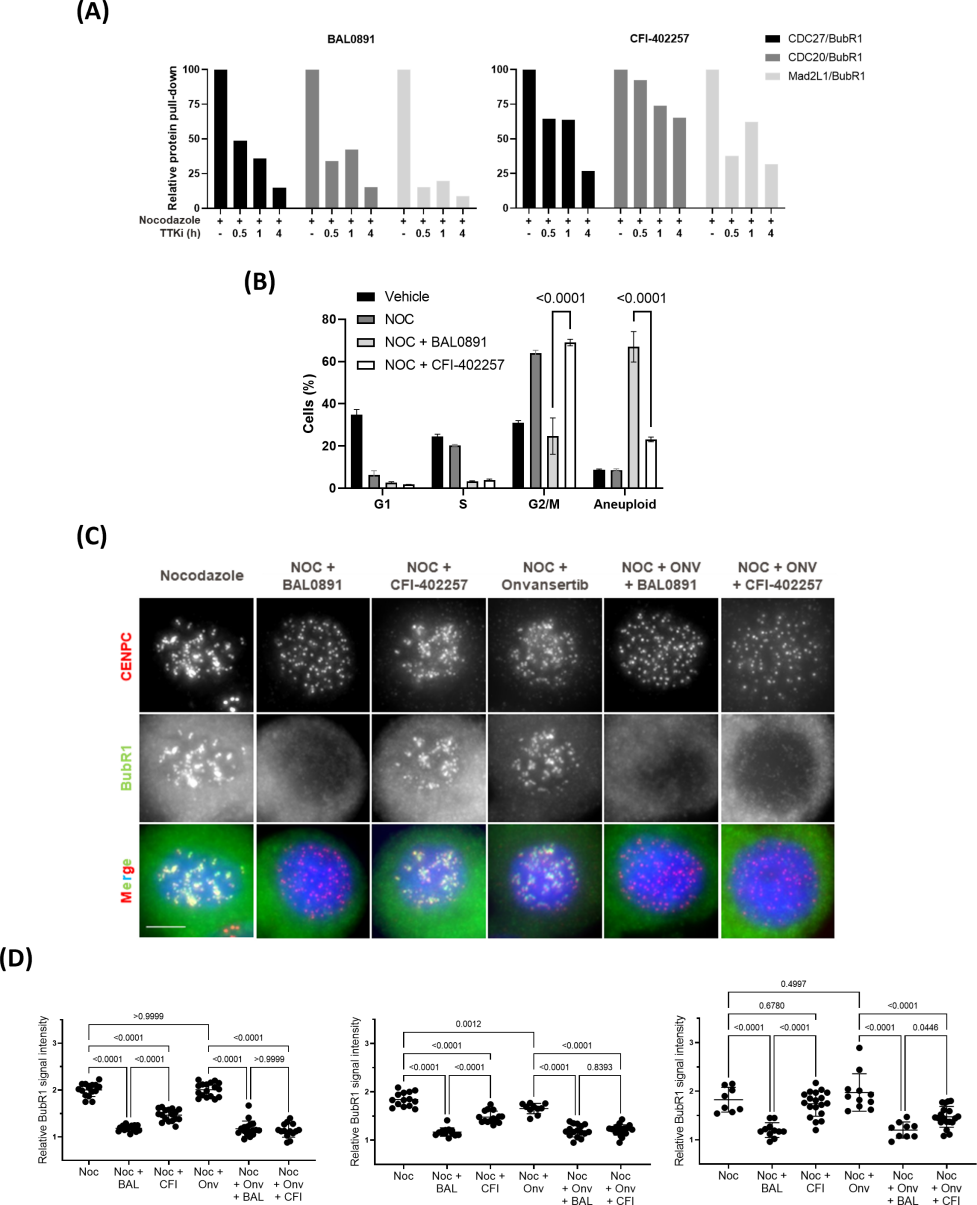
Efficient SAC disruption and mitotic exit associated with dual TTK and PLK1 inhibition. **(A)** Quantification of SAC protein interaction (relative protein pull-down) in HT29 cells blocked in mitosis with nocodazole (50 ng/mL, 18 h) and treated with GI_50_ BAL0891 or CFI-402257 for the indicated times. Interaction was quantified by dividing the intensity of the prey protein by the bait protein (BubR1) from immunoblot analyses. **(B)** % cells in G1, S and G2/M cell cycle phases and with higher ploidy (aneuploid) from flow cytometry profiles of HT29 cells blocked in mitosis with nocodazole (NOC, 100 ng/mL, 7 h) and treated with GI_50_ BAL0891 or CFI-402257 for 18 h. Data are presented as the mean ± SEM; n = 3 independent experiments. Statistics: two-way ANOVA with Tukey *post-hoc* for multiple comparisons. **(C)** Immunostaining of CENPC (red), BubR1 (green) and DNA (DAPI, blue) in HT29 cells blocked in mitosis with nocodazole (NOC, 100 ng/mL, 7 h) and then treated with GI_50_ BAL0891, CFI-402257 and/or onvansertib (ONV) for 1 h. Scale bar: 5 μm. **(D)** Quantification of BubR1 staining intensity at KTs from 3 independent experiments; 8-19 cells/condition were evaluated in each experiment. BAL0891 (BAL), CFI-402257 (CFI), onvansertib (Onv), nocodazole (Noc). Statistics: Brown-Forsythe and Welch ANOVA tests.

Despite no evidence of a mitotic block with BAL0891, and based on the observation that the role of PLK1 on the SAC is masked by its other roles in mitosis (14), it seemed relevant to pursue the hypothesis that a dual action on both TTK and PLK1 may allow BAL0891 to more efficiently disrupt the SAC as compared to an inhibitor, such as CFI-402257, that targets TTK but not PLK1 (36). The kinetics of SAC disruption using the half-maximal growth inhibitory concentration (GI_50_) of BAL0891 and CFI-402257 were, therefore, tested using the BubR1 SAC co-immunoprecipitation approach on nocodazole treated HT29 cells (**Figure 2A**). With both compounds, SAC breakage was observed after 30-60 minutes incubation. However, effects were more profound with BAL0891 which, unlike CFI-402257, reached almost total (>80-90%) loss of SAC complexes after 4 hours treatment. These observations were consistent with a more profound release from G2/M, concomitant with more substantial increases in aneuploidy associated with BAL0891 treatment as compared to CFI-402257 over a prolonged incubation period (**Figure 2B**). Taken together, these data indicate a higher potency of BAL0891 on SAC integrity as compared to the TTK-specific inhibitor CFI-402257.

In order to evaluate this hypothesis further, using a more quantitative assessment of SAC accumulation at the kinetochore (KT), an immunofluorescence assay was established. Using this assay, co-localization of BubR1 (as a marker of the SAC) with the KT protein CENPC could be directly visualized (**Figure 2C**). Specifically, treatment of nocodazole-blocked mitotic HT29 cells for 1 hour with a GI_50_ BAL0891 concentration resulted in a highly reproducible and significant (p<0.0001; **Figure 2D**) reduction in KT-associated BubR1 (**Figure 2C**). Some reduction was observed with GI_50_ CFI-402257 under the same conditions but the effects were not as potent as those observed with BAL0891 and did not always reach significance. Moreover, minor effects were observed for onvansertib. Strikingly, combined treatment with CFI-402257 and onvansertib reproduced the effect obtained with BAL0891 alone (**Figures 2C, D**). These data strongly support the conclusion that dual inhibition by BAL0891 of both TTK and PLK1 provides for a more efficient SAC disruption, which could have implications for anticancer potency as a monotherapy.

### 3.4 BAL0891 elicits potent, single agent antitumor activity in TNBC mouse xenograft models

Evaluation of the anti-proliferative activity of BAL0891 across tumor cell lines, derived from breast, endometrial, bladder, colorectal, gastric and renal cancers, indicated a potentially broad anticancer potential (**Supplementary Figure 2**) with GI_50_s of sensitive lines in the low nM range. Interestingly, evaluation of the anti-proliferative effects of BAL0891 in non-immortalized cells (HS68 human early passage fibroblasts and primary human mammary epithelial cells) indicated minimal activity, with GI_50_s >5 μM (not shown). Of note, in all tumor types differential responses were observed, with some models showing a minimal response. As an example, details of activity in TNBC lines are shown in **Supplementary Table 2**.

**Figure 3:**
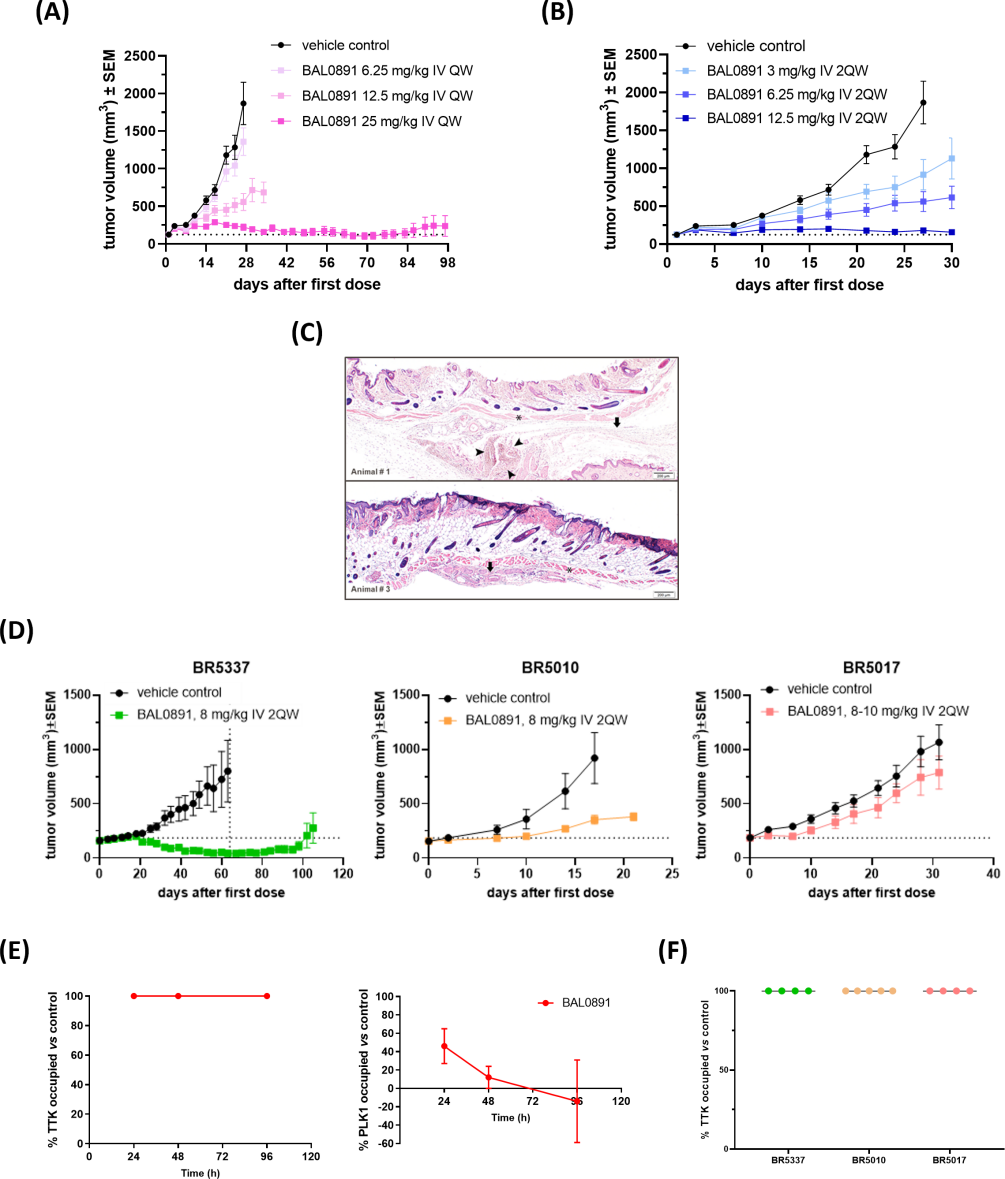
BAL0891 elicits potent, single agent antitumor activity *in vivo.* **(A)** MDA-MB-231 tumor-bearing animals treated with vehicle or BAL0891, administered IV QW as indicated. Mean tumor volume ± SEM; n = 8/group. **(B)** MDA-MB-231 tumor-bearing animals treated with vehicle or BAL0891, administered IV 2QW as indicated. Mean tumor volume ± SEM; n = 8/group. **(C)** H&E of skin/surrounding tissue from animals 1 and 3, from the site of tumor implantation from tumor-free animals treated with 25 mg/kg BAL0891 IV QW as shown in **(A)**. Pictures show skin, *panniculus carnosus* (asterisk) and subcutaneous *fascia* (arrow). Small aggregates of polygonal cells (arrowheads) are present which are most likely consistent with macrophages. **(D)** Representative graphs of TNBC PDX models with differential responses to BAL0891. Tumor-bearing animals treated with vehicle or BAL0891, IV 2QW as indicated. For model BR5017, dosing was initiated at 10 mg/kg and reduced to 8 mg/kg from day 15. Dashed vertical line indicates end of treatment; n = 4 or 5/group. Non-drug related animal deaths: one in the BAL0891 group (day 33) of model BR5337 and one in the vehicle group (day 54) of model BR5337. Drug-related animal deaths: one in the BAL0891 group (day 8) of model BR5017. **(E)** TTK and PLK1 drug occupancy in MDA-MB-231 tumors. BAL0891 was dosed at 12.5 mg/kg IV 2QW and tumors were collected for analysis 24, 48 and 96 h after 11 treatments (n = 2-3/time point). % drug-occupied TTK (left) and PLK1 (right) was *versus* vehicle control samples. **(F)** TTK drug occupancy in vehicle- or BAL0891-treated tumors obtained from **(D)** 4 h after the last treatment (n = 4 or 5/group). % drug-occupied TTK was *versus* vehicle control samples.

In order to establish optimal dosing schedules for animal tumor models, BAL0891 was extensively evaluated in the TNBC xenograft model MDA-MB-231 using weekly (QW, **Figure 3A**) and twice-weekly (2QW, **Figure 3B**) intermittent intravenous (IV) dosing schedules. For both schedules, BAL0891 was administered at the MTD and fractions thereof. All treatments were tolerated (**Supplementary Table 3**) with no drug-related animal deaths recorded. Twenty-seven days after treatment initiation, both QW and 2QW schedules showed dose-dependent antitumor activity; with MTD-dosing associated with tumor stasis and some regressing tumors (**Supplementary Table 3**). Moreover, as QW dosing could be administered longer, due to tail vein preservation, the QW MTD group was dosed until day 97, followed by an observation period until day 125. At this time point, two mice had no palpable tumor. These animals were, therefore, investigated for the presence of residual tumor cells by histopathological analysis of the tumor implantation site and surrounding tissue/skin. Both mice showed no detectable residual tumor cells (**Figure 3C**). Hence, 25% of treated mice could be considered as tumor-free (cured), an observation confirmed in a second independent experiment (data not shown).

BAL0891-monotherapy was further evaluated across a panel of PDX TNBC models. 2QW IV dosing was selected as it results in similar anticancer activity as QW during the treatment period and is more representative of a QW schedule in humans. Dosing was adjusted to 8 mg/kg for consistency across the screen, based on tolerability in the different mouse strains used for the screen and inclusion of some cachexic models. In some BAL0891 resistant models, a slightly higher dose of 10 mg/kg was assessed. Although cachexic tumor models showed some body weight losses, in general BAL0891 treatment was well tolerated (**Table 1**) and elicited a varied range of antitumor responses (**Figure 3D**). Specifically, of the thirteen TNBC models tested, five exhibited a ΔT/C (treated/control tumors) of <0.4, and were thus considered sensitive, including three models with tumor regressions (**Table 1**). Models with a ΔT/C of >0.4 to 0.6 were considered intermediate responders, while those with a ΔT/C >0.6 were considered minor responders. These data, in agreement with *in vitro* profiling (**Supplementary Table 2**), indicate a differential activity profile of BAL0891, with potent anticancer activity in a subset (~40%) of the TNBC PDX models assayed. This differential response profile could provide a basis for further response biomarker evaluation.

**Table 1:** Efficacy and tolerability of BAL0891 across a panel of thirteen TNBC PDX models.

Model ID	Assessment day	Efficacy ΔT/C	Tolerability ΔT/C	Regressions (%)	Response Category
BR5337	63	-0.18	1.01	74	Sensitive
BR10014	52	-0.2	0.86	46/69 (day 83)	Sensitive
BR5011	38	0.10	0.93	11*	Sensitive
BR5010	17	0.26	0.88		Sensitive
BR5013	60	0.29	0.97		Sensitive
BR1474	30	0.45	0.93		Intermediate
BR10582	24	0.48	0.97		Intermediate
BR1458	12	0.52	0.99		Intermediate
BR10539	56	0.52	1.06		Intermediate
BR5399	63	0.55	0.93		Intermediate
BR1282	15	0.66	0.98		Minor
BR5017	31	0.68	0.99		Minor
BR2014	20	0.87	0.91		Minor

BAL0891 (8 mg/kg) or vehicle was administered IV 2QW (n = 4-5/group). ΔT/C ([mean(T)-mean(T0)]/[mean(C)-mean(C0)]) were calculated on the day the first animal was removed due to large tumor size. For efficacy, the mean ΔT/C was based on the difference in tumor volume, for tolerability, on the fold-change in mean body weight. Non-drug-related animal deaths in the BAL0891 dosing group: one death in BR5337 (day 33), BR5013 (day 5) and BR2014 (day 20). Drug-related animal deaths in the BAL0891 dosing group: one in BR10014 (day 35), BR1458 (day 8), and BR5017 (day 8). Non-drug-related animal deaths in the vehicle group: one in BR5337 (day 52), BR5013 (day 32), BR5399 (day 30) and BR2014 (day 20). Regressions (% regression calculated from the median of the BAL0891 treatment group) represent a shrinkage of the tumor below the starting tumor size. *Regressions were observed in 2/5 mice and data from only these two mice are shown. Response category: Sensitive = efficacy ΔT/C <0.4; Intermediate = ΔT/C >0.4 to 0.6; Minor = ΔT/C >0.6.

### 3.5 Tumor drug accessibility does not explain tumor response

In order to assess tumor target occupancy, BAL0891-unoccupied TTK and PLK1 were evaluated in tumors obtained from treated mice. Mice harboring MDA-MB-231 tumors were treated IV 2QW with MTD BAL0891 and sacrificed 24, 48 and 96 hours after the last treatment. Consistent with *in vitro* target residency and occupancy data (**Figures 1A–C**) analysis for tumor TTK and PLK1 target occupancy showed that TTK was fully occupied by BAL0891 for ≥96 hours after the last dose, whereas PLK1 was only partially occupied (46%) at 24 hours and almost completely unoccupied after 48 and 96 hours (**Figure 3E**). BAL0891’s prolonged tumor TTK target residency was confirmed in two additional experiments (**Supplementary Figure 3**). Hence, the promising single agent activity observed with intermittent IV schedules is associated with prolonged tumor TTK target occupancy combined with transient PLK1 occupancy.

To assess whether anticancer responses to BAL0891 in the TNBC PDX models are related to differences in target accessibility, the same assay was performed using samples obtained from three models showing differential sensitivity to BAL0891 treatment (**Figure 3D**). In all cases, complete TTK target occupancy was observed in tumors obtained 4 hours after the last treatment (**Figure 3F**) indicating that dependency of the tumor on the target rather than drug availability is important for anticancer activity.

### 3.6 BAL0891 synergizes with paclitaxel and carboplatin in mouse TNBC models

**Figure 4:**
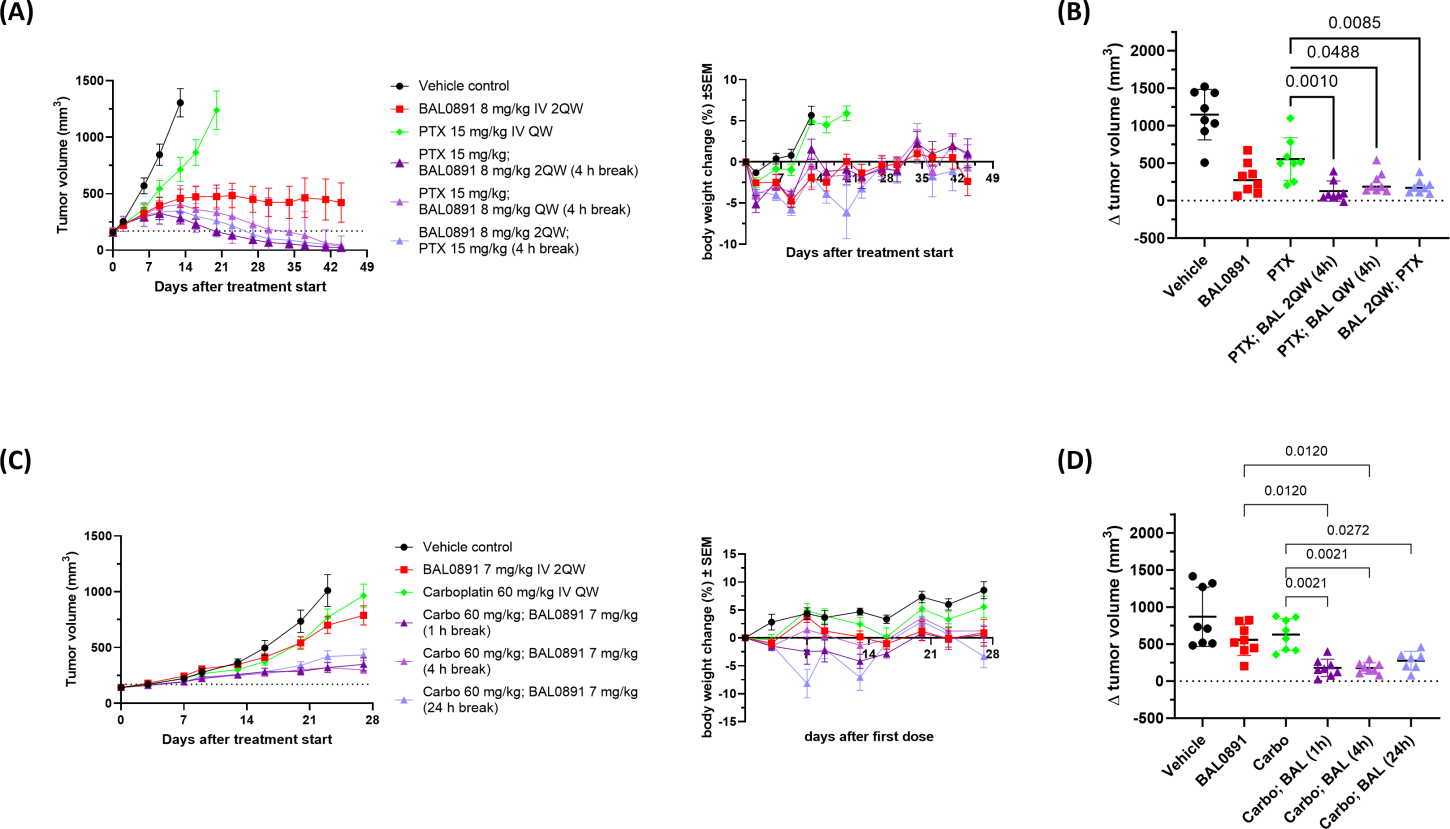
Synergistic efficacy of BAL0891 in combination with paclitaxel (PTX) or carboplatin (Carbo). **(A)** PTX (15 mg/kg) was administered IV QW to BR1282 PDX-bearing mice. BAL0891 (8 mg/kg) was administered IV 2QW or QW (4 h before or after PTX, when administered on the same day). Mean TV ± SEM (left) and mean BW changes in % ± SEM (right) are shown (n = 8/group). Drug-related animal deaths: one in the PTX/BAL0891 2QW combination group (day 21). **(B)** ΔTV (TV on day 16 - TV on day 0) of vehicle, 8 mg/kg BAL0891 IV 2QW, 15 mg/kg PTX IV QW or a combination of paclitaxel and BAL0891 (PTX; BAL) from the efficacy study in **(A)**. The line represents the mean ± SD. Statistical analysis: one-way-analysis of variance (1WA) with Holm-Sidak *post-hoc* for group comparisons. **(C)** Carbo (60 mg/kg) was administered IV QW to SK-OV-3 tumor-bearing mice. BAL0891 (7 mg/kg) was administered IV 2QW (1, 4 or 24 h after carbo, when administered on the same day). Mean TV ± SEM (left) and mean BW changes in % ± SEM (right) are shown (n = 8/group). Drug-related animal deaths: one in the 24 h break combination group (day 8). **(D)** ΔTV (TV on day 23 - TV on day 0) of vehicle, 7 mg/kg BAL0891 IV 2QW, 60 mg/kg carboplatin IV QW or a combination of carboplatin and BAL0891 (Carbo; BAL) from the efficacy study in **(C)**. The line represents the mean ± SD. Statistical analysis: one-way-analysis of variance (1WA) with Holm-Sidak *post-hoc* for group comparisons.

The chemotherapeutic agent paclitaxel is SoC for the treatment of patients with advanced TNBC (38). Hence, the antitumor activity of BAL0891 in combination with paclitaxel was evaluated in the TNBC PDX BR1282 model, which was selected due to its intermediate response to both paclitaxel and BAL0891 monotherapies (**Figure 4A**; **Supplementary Table 4**). Based on a prior tolerability study, 8 mg/kg BAL0891 (IV, 2QW) could be combined with a standard paclitaxel dosing regimen (15 mg/kg, IV, QW). In a first experiment, paclitaxel was administered first, and 4 or 24 hours later mice were treated with BAL0891 (when both compounds were administered on the same day). As expected, the single agents elicited a slowing of tumor growth while, strikingly, both combination regimens led to tolerated, tumor regressions associated with ~40% pathologically confirmed tumor-free animals (cures) (**Supplementary Table 4**).

A second study confirmed the synergistic interaction (see examples in **Figure 4A** and full data summary in **Supplementary Table 4**) and included additional combination groups assessing a shorter gap (2 hours) between paclitaxel and BAL0891 dosing, QW dosing of BAL0891 and the reverse order of administration (applying BAL0891 4 hours before paclitaxel). Independent of dosing regimen, 100% tumor regressions were observed. All five combination regimens were similarly efficacious, with 50-88% tumor-free animals, although the combination group with a 24-hour gap between treatments was slightly less well tolerated. Of note, despite 8 mg/kg BAL0891 being considered a relatively low dose for a QW schedule (~1/3 MTD), 50% cures were also observed in this combination group (**Supplementary Table 4**). Moreover, individual tumor volume change (Δ tumor volume) on the day the vehicle control group was euthanized due to tumors ≥1500 mm^3^, showed that tumor sizes in all the combination groups were significantly lower in comparison to paclitaxel single agent (p<0.05 - 0.001) (**Figure 4B**). These results strongly support the approach of combining BAL0891 with paclitaxel for the treatment of TNBC patients and demonstrate the potential for flexibility in the timing and order of drug administration, as well as the potential for low dose BAL0891 treatment.

Carboplatin is SoC in a number of tumor indications, including TNBC and ovarian cancer (38). Based on a prior tolerability study, 7 mg/kg BAL0891 (IV, 2QW) could be combined with a standard carboplatin dosing regimen (60 mg/kg, IV, QW). In the above BR1282 TNBC model, carboplatin monotherapy was without activity and BAL0891 combined with carboplatin showed an additive antitumor effect only when carboplatin was administered first. As carboplatin is a relevant treatment in ovarian cancer, the SK-OV-3 ovarian cell-derived xenograft (CDX) model, where both monotherapies induced a minor antitumor response, was further investigated using the preferred schedule (**Figure 4C**; **Supplementary Table 5**). Carboplatin was administered first, and 1, 4 or 24 hours afterwards the mice were treated with BAL0891 (when both compounds were administered on the same day). Furthermore, QW dosing of BAL0891 administered 4 hours after carboplatin was also evaluated. As expected, the single agents showed only a slowing of tumor growth while the combination regimens with 2QW BAL0891 dosing led to tumor stasis. In contrast to observations with paclitaxel, QW dosing with low-dose BAL0891 (administered 4 hours after carboplatin) showed only a small improvement in efficacy (**Supplementary Table 5**). Individual Δ tumor volumes showed that the tumor sizes in the combination groups with a 1 and 4 hour gap between treatments, were significantly lower in comparison to BAL0891 and carboplatin single agent (p<0.012 - 0.0021) (**Figure 4D**) and synergy was confirmed for these combination groups (CCI: -0.26). The combination group with the 24 hour gap only showed significance versus the carboplatin group (p=0.0272). Moreover, in the 1 and 4 hour gap groups, 1 of 8 mice showed pathologically confirmed tumor-free animals (cures) (**Supplementary Table 5**). In all cases, the combinations were tolerated, with the 24-hour gap associated with reduced tolerability (**Figure 4C**). These results support combining BAL0891 with carboplatin, using a treatment regimen where carboplatin is administered first and a relatively short gap between drug administrations.

## 4 Discussion

In this study, we report a novel, small molecule, dual TTK/PLK1 mitotic checkpoint inhibitor known as BAL0891. As far as we are aware, the mechanism of action (MoA) of BAL0891, associated with a prolonged inhibition of TTK and a transient effect on PLK1, is unique to this molecule. Hence, we suggest that BAL0891 represents a first-in-class TTK/PLK1 inhibitor for the treatment of cancer patients. The data presented here indicate that inhibition of both TTK and PLK1 contributes to a more efficient and rapid disruption of the SAC, potentiating aberrant mitotic exit as compared to TTK-specific inhibitors. Contrary to observations with potent PLK1-specific inhibitors (39), no indication of a mitotic block is evident, suggesting a dominant contribution of TTK inhibition to the MoA of BAL0891. Unmasking of PLK1’s role at the SAC (13, 14), together with a potential bypass of other mitotic defects caused by transient PLK1 inhibition, could beneficially contribute to the anticancer activity of BAL0891. In this regard, potent single agent activity has been demonstrated in a number of mouse models of human TNBC, using intermittent dosing schedules, in some cases accompanied by tumor regressions and tumor-free animals. This is in contrast to TTK-specific inhibition, which is associated with moderate single agent activity even with daily dosing, characterized by a slowing or stabilization of tumor growth rather than prolonged regressions (36, 40–43). For example, although one should compare data from different experiments with caution, daily dosing with CFI-402257 of MDA-MB-231 xenografted mice has been reported to elicit a maximum tumor growth inhibition of 89%, with no apparent tumor regressions reported (36). This is in contrast to the data presented here, with 25% tumor-free animals following optimal weekly BAL0891 dosing. Another important aspect, based on clinical data from PLK1 inhibitors explored as monotherapies in cancer patients, is the limited therapeutic effects reported in patients with solid tumors due to dose-limiting toxicities (17, 19, 44). BAL0891 is only transiently affecting PLK1 activity and does not block mitotic progression. As the latter is postulated to augment the negative side effects in highly proliferative, non-tumor tissues associated with PLK1-specific inhibitors (45–47), it will be of interest to see if BAL0891s unique kinase inhibition profile and potential for intermittent dosing will lead to a favorable therapeutic index in cancer patients. Specifically, it is tempting to postulate that BAL0891s unique profile could potentially alleviate the primary dose-limiting toxicity associated with PLK1-and TTK-specific inhibitors; namely hematologic toxicity (including neutropenia) (19, 48).

*In vitro* and *in vivo* investigation of the breadth of anticancer activity of BAL0891 in a panel of TNBC models has indicated a differential response to BAL0891 treatment, with a subset (~40%) of *in vivo* tumor models responding well to treatment, as well as clearly refractory models. Differential antitumor responses have also been observed in PDX models derived from other tumor histotypes (including gastric and hepatocellular cancer). These data suggest that patient selection could be an important aspect to consider for optimizing the clinical development of agents targeting both TTK and PLK1. Presently, limited data is available on potential tumor response markers for TTK-specific inhibitors. Based on available clinical information, it appears that TTK inhibitors have been mainly investigated in solid tumors and breast cancer subtypes, like TNBC, but patients have not been otherwise selected (17). It is of note that recently a clinical trial was initiated in which CFI-402257 is being investigated as a single agent and in combination with fulvestrant in CDK4/6 inhibitor-resistant advanced estrogen receptor positive breast cancer associated with loss of the tumor suppressor, cell cycle regulator retinoblastoma (Rb) (NCT05251714). The lack of Rb mutated models in our screens, unfortunately, hinders any assessment of the relevance of Rb status with regard to response to BAL0891. However, based on the publication of Anderhub et al, 2019, who evaluated potential response criteria across tumor cell lines from different histotypes (including TNBC) *in vitro*, some conclusions can be made. Specifically, the hypothesis that susceptibility to the TTK-specific inhibitor BOS172722 is associated with more rapidly dividing cells was not confirmed for BAL0891 in either the TNBC cell line or PDX screens. Indeed, doubling time *in vitro* and PDX tumor growth rates (together with Ki67 proliferation marker status at baseline) did not correlate with response. Moreover, taking the PDX screen as the more relevant approach, the mutated genes postulated by Anderhub et al. to be enriched in sensitive tumor cell lines could not be confirmed in our study (data not shown). These points, together with the potent efficacy associated with BAL0891 monotherapy, support the conclusion that the MoA of BAL0891 is differentiated from TTK-specific inhibitors.

Interestingly, clinical trials in solid tumor patients with first-generation PLK1 inhibitors did not select for specific patient populations, which could partly explain the modest clinical benefit observed (17, 19). Based on the identification of genes, such as RAS (a key signal transduction GTPase) and BRCA1 (a tumor suppressor involved in DNA damage repair) which show synthetic lethality with PLK1 inhibition (20, 49), the next generation PLK1 inhibitor onvansertib is currently being assessed in KRAS-mutated metastatic colorectal carcinoma patients in combination with FOLFIRI and bevacizumab, with promising overall response rates recently reported (50). The low level of RAS mutation in breast cancer, reflected in our screening models, does not allow us to appropriately address a connection between RAS status and response. Additionally, with regard to BRCA status, no BRCA1 or BRCA2 mutations with known clinical relevance in TNBC patients were found.

It is highly likely, based on the novel MoA of BAL0891 and the discussion above, that tumor response biomarkers will differ from those postulated for TTK- and PLK1-specific compounds. A more extensive BAL0891 PDX screen would be required in order to confirm the potential predictive value of any response biomarker(s). In this context, complementary to evaluation of any link between BAL0891 response and gene mutation status, there could also be value in a response signature based on gene expression. Indeed, previous data indicating a potential to predict CFI-402257 activity in TNBC cell lines based on a two-gene expression signature involving components (ANAPC4 and CDC20) of the anaphase-promoting complex, which promotes mitotic progression following inactivation of the SAC, is supportive of such an approach (51).

Currently, predictive criteria relevant to BAL0891 response in TNBC models are not available. However, work focussed on the elucidation of potential response biomarkers using tumor material obtained from the PDX models used in our studies has been initiated. With reference to previous studies using TTK- and PLK1-specific approaches (discussed above), and based on the MoA of BAL0891, loss of tumor suppressors, aberrant signal transduction/cell cycle modulation or even alterations in genes involved in mitotic progression itself may also have implications for tumor response to dual TTK/PLK1 inhibition. Hypotheses worthy of further exploration.

The modest activity of TTK-specific inhibitors as single agents has led to the investigation of these small molecules in preclinical combination studies, with particular emphasis on taxanes and TNBC (40, 43, 52, 53). Indeed, some have entered clinical trials, although a tight therapeutic index in combination with paclitaxel has been reported (54, 55). This combination strategy exploits the ability of taxanes to stabilize microtubule dynamics (impeding mitotic progression) while TTK inhibitors induce aberrant mitotic exit through SAC override, ultimately causing enhanced tumor cell death. The enhancement of paclitaxel drug sensitivity associated with TTK knockdown in TNBC models (56) and the role of PLK1 in TNBC cell-regrowth following paclitaxel treatment (57) further supports this approach. To the best of our knowledge, this is the first report of a high frequency of tumor-free animals (pathologically confirmed as cures) after treatment with tolerated combinations of a TTK inhibitor with paclitaxel, in a TNBC PDX model with limited response to either agent as a monotherapy. Furthermore, flexibility in the timing and order of administration of BAL0891 with paclitaxel, as well as clear synergy also with a sub-MTD weekly BAL0891 dose, are parameters of great interest for safe clinical evaluation in cancer patients where treatment with paclitaxel is SoC.

Consistent with previous *in vitro* evaluations (53, 58), synergy is also reported here for the first time with carboplatin administered prior to BAL0891 using an ovarian cancer xenograft model. These data suggest that prior DNA damage could accentuate chromosomal defects caused by aberrant mitotic progression of genetically unstable tumor cells through the action of BAL0891, supporting the utility of this combination for the treatment of cancer indications where carboplatin treatment is indicated. It is also tempting to speculate, based on the postulated role of both PLK1 and TTK in DNA damage response (see (59, 60), and references therein) that persistent DNA damage after combined treatment with carboplatin and BAL0891 might contribute to increased tumor cell death.

**Figure 5:**
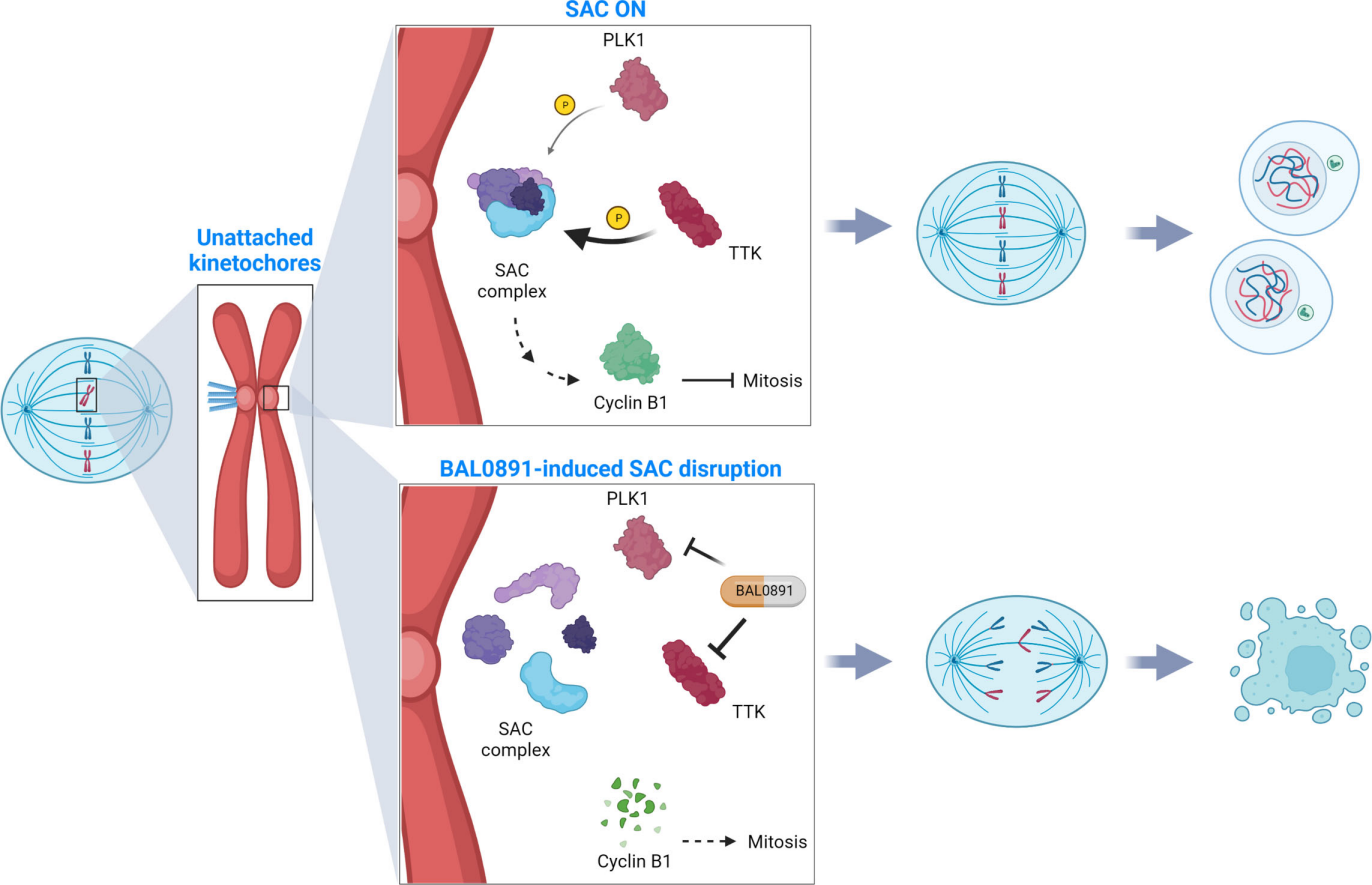
SAC deactivation by BAL0891 causes aberrant tumor cell division. TTK and PLK1 collaborate in activating the mitotic spindle assembly checkpoint (SAC) at the kinetochore, ensuring correct chromosome alignment and segregation prior to mitotic exit (SAC ON). BAL0891-mediated prolonged inhibition of TTK combined with a transient effect on PLK1 leads to a rapid disruption of the SAC, leaving tumor cells without adequate time for correct chromosome segregation (BAL0891-induced SAC disruption) contributing to a potent anticancer activity. (Figure created with BioRender.com).

Taken together, these results suggest that the unique TTK/PLK1 selectivity and MoA of BAL0891 (**Figure 5**) contributes to a potent single agent activity in a subset of TNBC models. Moreover, combination approaches may provide a potential to expand responsive patient populations. Hence, BAL0891 is a promising small molecule, kinase inhibitor worthy of clinical evaluation. Indeed, a Phase 1 program in cancer patients with solid tumors, investigating intermittent BAL0891 dosing, has recently been initiated (NCT05768932).

## Data Availability

The original contributions presented in the study are included in the article/**Supplementary Material**. Further inquiries can be directed to the corresponding author.
