# Supplementary material for: Dual TTK/PLK1 inhibition has potent anticancer activity in TNBC as monotherapy and in combination
Source: Front Oncol. 2024 Aug 9;14:1447807. doi: 10.3389/fonc.2024.1447807 (PMC11341980; doi:10.3389/fonc.2024.1447807)
Supplement: Supplementary file 1 [file DataSheet_1.docx]

Supplementary Material

# Supplementary Materials and Methods

## Target residency and occupancy evaluation

TTK kinase domain was immobilized on a Ni-NTA sensor chip with His-tag capturing and amine-coupling to a level of 4000–6000 RU. Biotinylated PLK1 (Carna Biosciences) was captured on a streptavidin sensor chip with a targeted immobilization level of 4000 RU. BAL0891 binding was measured in binding buffer (10 mM Tris, 10 mM MgCl_2_, 0.01% Tween-20, 1 mM DTT at pH 6.8) with 1% (v/v) DMSO using single cycle kinetics by injecting an increasing concentration range of 1 – 3.16 – 10 – 31.6 – 100 nM. Flow rate was 30 μL/min, and association time per injection was 100 s. Following the last injection, dissociation was monitored for at least 30 min with no regeneration. The buffer injection and the reference channel signals were subtracted (double referencing) from the BAL0891 signal. Resulting data were fit, using the Biacore Evaluation software, to the simple 1:1 Langmuir binding model. All kinetic constants were within the working range of the Biacore T200. To determine the reliability of the curve fit, standard Biacore checks were applied as previously outlined (Willemsen-Seegers et al, J Mol Biol. 2017 Feb;429(4):574–86).

For TTK target occupancy evaluation, cell or tumor samples were homogenized in TPER lysis buffer (Thermo Fisher Scientific, #78510) supplemented with a protease inhibitor cocktail (Thermo Fisher Scientific, #78429) and centrifuged. The supernatant was incubated 1 h with a probe containing a TTK-specific inhibitor linked to a biotin moiety, followed by incubation with Streptavidin beads (VWR, Pierce, # PIER88817) for 30 min. After washing the beads, the captured TTK protein was denatured with 50 μM DTT and 4 M urea. After incubation at 55°C for 15 min, 35 mg/mL iodocetamide (Sigma, #I1149-5G) was added and the sample was incubated for 15 min. To digest the TTK protein into peptide fragments, 20 mg/mL trypsin (Sigma, #T8253) was added, incubated at RT overnight (O/N) and stopped with 2% FA. The resulting solution was analyzed for the presence of two specific TTK tryptic peptides (#1/2) that have the following sequences: VFQVLNEK/NKTESSLLAK. For PLK1 target occupancy evaluation, a similar protocol was used except that a probe containing a PLK1 specific inhibitor linked to a biotin moiety was used to capture unoccupied PLK1. Two specific PLK1 tryptic peptides (#1/8) have the following sequences: HINPVAASLIQK/LSLLEEYGC[CAM]C[CAM]K.

## Immunoblotting

Whole-cell lysates were generated using cell lysis buffer (20 mM Tris HCl pH 7.5, 150 mM NaCl, 1 mM EGTA, 1 mM EDTA, 1% [v/v] NP40, 1% [v/v] Triton-X, 10 μL/mL protease and phosphatase inhibitor cocktail [Thermo Fisher Scientific, #78441], 1 mM PMSF [Fluka, #93482]). BCA assay was used to evaluate protein concentrations (Pierce, #22660). SDS–PAGE gels were transferred onto 0.2 μM PVDF membranes using the Trans-Blot Turbo Transfer Pack (mixed molecular weight). Membranes were blocked with 3% BSA (w/v) in TBST and subsequently probed with primary antibodies diluted in 3% BSA/TBST O/N at 4°C. After incubation with secondary antibodies (anti-rabbit-IgG-HRP #A0545 or anti-mouse-IgG-HRP #A4416, Sigma- Aldrich) at 1:5000 dilution for 1 h at RT, membranes were developed with ECL prime detection reagent and images obtained using a Fusion S imager (Witec AG).

## Co-immunoprecipitation SAC assay

Co-immunoprecipitation experiments were performed using Protein G Sepharose fast flow (Sigma, #P3296-5ML). 500 - 800 μg of fresh protein lysate was prepared in a total volume of 500 μL cell extraction buffer (50 mM HEPES pH 7.4, 150 mM NaCl, 5 mM EGTA, 1 mM EDTA, 1% [v/v] NP40, 10 μL/mL protease and phosphatase inhibitor cocktail [Thermo Fisher Scientific, #78441], 1 mM DTT, 1mM PMSF [Fluka, #93482]). 0.5 - 1 μg of BubR1 antibody (BD Biosciences) was added with end-over-end-mixing at 4°C O/N, followed by incubation with 25 μL of Protein G Sepharose with end-over-end-mixing for 3 h at 4°C. After extensive washing, samples were centrifuged in a microfuge at 4°C at maximum speed for 30 sec, immunoprecipitates/beads were resuspended in 30 μL of 2x Laemmli sample buffer and boiled at 95°C for 5 min followed by immunoblotting to detect protein–protein interaction. Quantitative densitometry analysis was carried out using the Evolution Capt software (Vilber Lourmat).

## Immunofluorescence microscopy

Cells were seeded on 13-mm glass coverslips in full medium and left to adhere O/N. After the appropriate treatments, cells were fixed with ice-cold 100% methanol for 5 min at -20°C and then rehydrated in PBS for 10 min. Coverslips were blocked for 30 min with 3% BSA (w/v) in PBS (BP) and then incubated with a 1:100 or 1:1000 dilution of BubR1 or CENP-C primary antibodies in BP, respectively, for 1 h in a moist environment. After washes with PBS, cells were incubated with fluorophore-labelled secondary antibodies (Life technologies, goat-anti-mouse AlexaFluor 647 #A-21236 and goat-anti-guinea pig AlexaFluor 568 #A-11075) at 1:1000 dilution in BP for 1 h. After washes with PBS, coverslips were mounted using SlowFade^®^ Diamond Antifade Mountant containing DAPI (Molecular Probes, #S36968). Images of randomly selected cells were acquired as 10 μm z-stacks with a step-size of 0.5 μm using the Nikon Eclipse Ti microscope. Images were projected (maximum intensity projection) using the NIS Elements AR software (Nikon) and quantification was performed using the General Analysis 2 tool. The quantification process comprised the following steps: selection of mitotic cells via intensity measurement of a threshold DAPI signal; generation of a kinetochore mask based on the intensity of bright spots (CENPC signal) inside the selected nuclei; generation of a tertiary mask for the BubR1 signal at kinetochores and BubR1 background measurements in the selected nuclei; export of reference images and signal intensities per cell.

## Cell growth assays

Cell lines were seeded in appropriate culture medium into 96-well plates and 9 serial compound dilutions (0.004 – 1 μM) were added 24 h later for 120 h. Residual cells were stained with crystal violet or YO-PRO (Molecular Probes, #Y-3603). GI_50_ values were calculated by fitting the data to a sigmoidal dose–response model with a variable slope using GraphPad Prism software after subtraction of the starting control signal. The GI_50_s used for SAC analyses were as follows: 14 nM BAL0891, 31-38 nM CFI-402257, 14 nM onvansertib.

# Supplementary Figures and Tables

**Supplementary Table 1. Screens of BAL0891 kinase selectivity.**

Residual kinase activity, expressed as % control, using 30 or 300 nM BAL0891. Kinases inhibited more >50% are shown. Screens were performed at Reaction Biology (top table) and Eurofins (bottom table) following their standard protocols. TTK: Threonine Tyrosine Kinase; PLK1, 3 and 4: Polo-Like Kinase 1, 3 and 4; TSSK1 and 4: Testis Specific Serine Kinase 1 and 4; TSF1 (STK16): Serine/Threonine Kinase 16; TNK1: Tyrosine Kinase Non Receptor 1; STK33, 17A (DRAK1) and 17B: Serine/Threonine Kinase 33, 17a and 17b; SNK: polo-like kinase 2; SAK: polo-like kinase 4; PKC-mu: Protein Kinase C Mu; NEK9: NIMA Related Kinase 9; MKNK2: MAPK Interacting Serine/Threonine Kinase 2; MELK: Maternal Embryonic Leucine Zipper Kinase; LRKK2 (wt, R1441C, I2020T and G2019S): Leucine Rich Repeat Kinase 2; JNK1, 2 and 3: Mitogen-Activated Protein Kinase 8, 9 and 10; DAPK2 and 3: Death Associated Protein Kinase 2 and 3; CHK2 (wt, R145W, I157T): Checkpoint Kinase 2; BUB1B: BUB1 Mitotic Checkpoint Serine/Threonine Kinase B; ZIPK: Death Associated Protein Kinase 3; ULK1: Unc-51 Like Autophagy Activating Kinase 1; PHKG2: Phosphorylase Kinase Catalytic Subunit Gamma 2; MNK2: MAPK Interacting Serine/Threonine Kinase 2; CAMKIIγ: Calcium/Calmodulin Dependent Protein Kinase II Gamma; ARK5: NUAK Family Kinase 1; ACK1: Tyrosine Kinase Non Receptor 2; AAK1: AP2 Associated Kinase 1.

| **Reaction Biology kinase assay** | **BAL0891** | |
| --- | --- | --- |
|  | **30 nM** | **300 nM** |
| **TTK** | 13 | 1 |
| **PLK1** | 48 | 10 |
| **TSSK4** |  | 6 |
| **TSSK1** |  | 30 |
| **TSF1 (STK16)** |  | 11 |
| **TNK1** |  | 25 |
| **STK33** |  | 29 |
| **STK17A (DRAK1)** |  | 6 |
| **SNK** |  | 26 |
| **SAK** |  | 25 |
| **PLK3** |  | 15 |
| **PKC-mu** |  | 31 |
| **NEK9** |  | 20 |
| **MKNK2** |  | 29 |
| **MELK** |  | 27 |
| **LRRK2 wt** |  | 31 |
| **LRRK2 R1441C** |  | 48 |
| **LRRK2 I2020T** |  | 42 |
| **LRRK2 G2019S** |  | 13 |
| **JNK3** |  | 24 |
| **JNK2** |  | 39 |
| **JNK1** |  | 40 |
| **DAPK3** |  | 30 |
| **DAPK2** |  | 30 |
| **CHK2 wt** |  | 21 |
| **CHK2 R145W** |  | 25 |
| **CHK2 I157T** |  | 26 |
| **BUB1B** |  | 43 |

| **Eurofins kinase assay** | **BAL0891** | |
| --- | --- | --- |
|  | **30 nM** | **300 nM** |
| **TTK** | -7 | -10 |
| **PLK1** |  | 9 |
| **ZIPK** |  | 38 |
| **ULK1** |  | 32 |
| **TSSK1** |  | 19 |
| **TSF1 (STK16)** |  | 46 |
| **STK17B** |  | 23 |
| **STK17A (DRAK1)** | 49 | 6 |
| **SNK** |  | 50 |
| **PLK4** |  | 12 |
| **PLK3** |  | 13 |
| **PHKG2** |  | 40 |
| **NEK9** | 34 | -1 |
| **MNK2** |  | 34 |
| **MELK** |  | 24 |
| **LRRK2 wt** |  | 38 |
| **JNK3** |  | 14 |
| **CAMKIIγ** |  | 47 |
| **ARK5** |  | 25 |
| **ACK1** |  | 48 |
| **AAK1** | 46 | 8 |

**Supplementary Figure 1. The dual TTK and PLK1 inhibitor BAL0891 causes aberrant mitotic exit while the PLK1-inhibitor onvansertib blocks cells in mitosis.**

1. Immunoblot analysis of HT29 extracts from cells blocked in mitosis with nocodazole (25 ng/mL, 18 h) and treated for 4 h with increasing concentrations of BAL0891. (p)TCTP: translational controlled tumor protein (PLK1 activity marker), pH3: phospho-histone H3.
2. Immunoblot analysis of SAC components in HT29 extracts from cells blocked in mitosis with nocodazole (50 ng/mL, 18 h) and then treated with BAL0891 (50 nM) for the indicated times.
3. Representative flow cytometry plots of HT29 cells blocked in mitosis with nocodazole (Noc, 100 ng/mL, 7 h) and treated with BAL0891 (50 nM) for 18 h.
4. Immunoblot analysis of HT29 extracts from cells blocked in mitosis with nocodazole (25 ng/mL, 18 h) and treated for 4 h with increasing concentrations of onvansertib. (p)TCTP: PLK1 activity marker.
5. Immunoblot analysis of HT29 cells blocked in mitosis with either nocodazole (50 ng/mL, 18 h) or onvansertib (200 nM, 18 h) and then treated with BAL0891 (50 nM) for 4 h. (p)TCTP: PLK1 activity marker, pH3: phospho-histone H3. GAPDH: loading control.
6. Quantification of cells (%) in G1, S and G2/M cell cycle phases and with higher ploidy (aneuploid) from flow cytometry profiles of HT29 cells blocked in mitosis with onvansertib (Onv, 200 nM, 7 h) and treated with BAL0891 (14 nM) for 18 h. A representative experiment is shown.

**Supplementary Figure 2. Anti-proliferative activity of BAL0891 across tumor cell lines.**

Maximal responses determined from concentration-response curves in cell lines treated with BAL0891 for 5 days. BC=breast cancer, including ER+ and HER2+ (black symbols) and TNBC (white symbols). EAC=endometrial cancer. UC=bladder cancer. CRC=colorectal cancer. GC=gastric cancer. RCC=renal cancer. Data were obtained using the crystal violet or YO-PRO cell growth assay. Medians are represented by a horizontal line. The red dashed line indicates 100% inhibition of tumor cell growth. Points above this line indicate tumor cell death.

**Supplementary Table 2. Anti-proliferative activity of BAL0891 in triple-negative breast cancer (TNBC) cell lines.**

GI_50_ concentration and maximal response (Amax) were determined from concentration-response curves in cell lines treated with BAL0891 for 5 days. The maximum concentration tested was 1000 nM.

| **Subtype** | **Cell Line** | **GI_50_ (nM)** | **A_max_ (%)** |
| --- | --- | --- | --- |
| **TNBC** | MDA-MB-468 | 33 | 108 |
|  | HCC70 | 19 | 96 |
|  | CAL-51 | 4 | 92 |
|  | MDA-MB-231 | 40 | 79 |
|  | BT549 | 20 | 79 |
|  | HCC1395 | 14 | 70 |
|  | Hs578T | 158 | 63 |
|  | CAL-120 | 587 | 58 |
|  | MDA-MB-453 | > 1000 | 32 |
|  | HCC1937 | > 1000 | 27 |

**Supplementary Table 3. Efficacy and tolerability of BAL0891 in the MDA-MB-231 tumor model.**

BAL0891 was administered IV QW or 2QW at the indicated concentrations. ΔT/C ([mean(T)-mean(T0)] / [mean(C)-mean(C0)]) were measured on the day the first animal was removed due to large tumor size. For efficacy, the mean ΔT/C was based on the difference in tumor volume, for tolerability, on the mean fold-change in body weight. Regressions (% regression calculated from the median of the BAL0891 treatment group) represent a shrinkage of the tumor below the starting tumor size. *Regressions were observed in 1/8 mice. **Regressions were observed in 2/8 mice. In groups where regressions were not seen in all mice, only data from the regressing mice are presented.

| **BAL0891** | **Efficacy**  **ΔT/C (day 27)** | **Tolerability**  **ΔT/C (day 27)** | **Regressions (%) (day 27)** | **Regressions (%)**  **(end of treatment day)** |
| --- | --- | --- | --- | --- |
| **6.25 mg/kg, QW IV** | 0.73 | 1.01 |  |  |
| **12.5 mg/kg, QW IV** | 0.30 | 0.95 | 15* |  |
| **25 mg/kg, QW IV** | 0.12 | 0.93 | 55* | 47 (97) |
| **3 mg/kg, 2QW IV** | 0.49 | 0.96 |  |  |
| **6.25 mg/kg, 2QW IV** | 0.30 | 0.95 | 89* |  |
| **12.5 mg/kg, 2QW IV** | 0.10 | 0.89 | 34** | 55 (34)* |

**Supplementary Figure 3. Tumor-derived TTK is drug occupied up to 6 days after administration of BAL0891.**

1. MDA-MB-231 tumor-bearing animals were treated with IV vehicle or 12.5 mg/kg BAL0891 on day 1, 4 and 8. n = 9 vehicle controls and n = 15 BAL0891.
2. TTK drug occupancy analysis in vehicle- or BAL0891-treated tumors obtained from A at the times shown after the last dose (n = 2/time point for vehicle and n = 3/time point for BAL0891-treated groups), The amount of drug-occupied TTK expressed as a % was calculated compared to vehicle control samples. The graphs represent data from two independent TTK representing tryptic peptides.
3. MDA-MB-231 tumor-bearing animals were treated with vehicle or 25 mg/kg IV BAL0891 on day 1. Due to some body weight loss, the second BAL0891 dose on day 9 was reduced to 20 mg/kg. n = 9 control and n = 15 BAL0891.
4. TTK drug occupancy analysis in vehicle- or BAL0891-treated tumors obtained from C at the times shown after the last dose (n = 3/time point). The amount of drug-occupied TTK expressed as a % was calculated compared to vehicle control samples. The graphs represent data from two independent TTK-representing tryptic peptides.

**Supplementary Table 4. Efficacy and tolerability results from two independent studies evaluating BAL0891 in combination with paclitaxel in the BR1282 PDX model.**

Paclitaxel (15 mg/kg) was administered IV QW to BR1282 PDX-bearing mice. BAL0891 (8 mg/kg) was administered IV QW or 2QW (2, 4 or 24 h after paclitaxel, when administered on the same day). When BAL0891 was administered first on the same day, paclitaxel was administered 4 h after. ΔT/C ([mean(T)-mean(T0)] / [mean(C)-mean(C0)]) were measured on the day the first animal was removed due to large tumor size. For efficacy, the mean ΔT/C was based on the difference in tumor volume, for tolerability, on the fold-change in mean body weight. Mice with a single measure of 20% BWL were culled. In Study 1, one non-drug-related death occurred in the BAL0891 single agent dosing group (day 21) and one mouse in the 24 h combination group was euthanized due to body weight loss > 20% (day 28). In Study 2, one mouse in the combination group PTX QW and BAL0891 2QW (4 h break) was euthanized due to body weight loss >20% (day 23). Regressions (% regression calculated from the median of the treatment group) represent a shrinkage of the tumor below the starting tumor size. *Regressions were observed in 1/8 mice. **Regressions were observed in 3/8 mice. ***Regressions were observed in 4/8 mice. In groups where regressions were not seen in all mice, only data from the regressing mice are presented.

**Study 1:**

| **Paclitaxel** | **BAL0891** | **Efficacy**  **ΔT/C (day 16)** | **Tolerability**  **ΔT/C (day 16)** | **Regressions (%) (end of treatment day)** | **Tumor-free (%) (end of observation period)** |
| --- | --- | --- | --- | --- | --- |
| **-** | **8 mg/kg, 2QW, IV** | 0.45 | 1.01 |  |  |
| **15 mg/kg, QW, IV** | **-** | 0.24 | 1.00 | 30 (37)* |  |
| **15 mg/kg, QW, IV** | **8 mg/kg, (4h) 2QW, IV** | 0.03 | 0.97 | 75 (37) | 37.5 |
| **15 mg/kg, QW, IV** | **8 mg/kg, (24h) 2QW, IV** | 0.02 | 0.95 | 100 (37) | 37.5 |

**Study 2:**

| **Paclitaxel** | **BAL0891** | **Efficacy**  **ΔT/C (day 13)** | **Tolerability**  **ΔT/C (day 13)** | **Regressions (%) (end of treatment day)** | **Tumor-free (%) (end of observation period)** |
| --- | --- | --- | --- | --- | --- |
| **-** | **8 mg/kg, (2h) 2QW, IV** | 0.32 | 0.96 | 51 (44)** |  |
| **-** | **8 mg/kg, (4h) 2QW, IV** | 0.26 | 0.93 | 28 (44)*** |  |
| **15 mg/kg, QW, IV** | **-** | 0.48 | 0.99 |  |  |
| **15 mg/kg, QW, IV** | **8 mg/kg, (4h) 2QW, IV** | 0.11 | 0.93 | 89 (44) | 75 |
| **15 mg/kg, QW, IV** | **8 mg/kg, (24h) 2QW, IV** | 0.17 | 0.91 | 77 (44) | 87.5 |
| **15 mg/kg, QW, IV** | **8 mg/kg, (2h) 2QW, IV** | 0.13 | 0.93 | 86 (44) | 87.5 |
| **15 mg/kg, QW, IV** | **8 mg/kg, (4h) QW, IV** | 0.21 | 0.96 | 89 (44) | 50 |
| **BAL0891** | **Paclitaxel** |  |  |  |  |
| **8 mg/kg, 2QW, IV** | **15 mg/kg, (4h) QW, IV** | 0.16 | 0.94 | 79 (44) | 62.5 |

**Supplementary Table 5. Efficacy and tolerability of BAL0891 in combination with carboplatin in the SK-OV-3 tumor model.**

Carboplatin (60 mg/kg) was administered IV QW to SK-OV-3 tumor-bearing mice. BAL0891 (7 mg/kg) was administered IV QW or 2QW (1, 4 or 24 h after carboplatin, when administered on the same day). ΔT/C ([mean(T)-mean(T0)] / [mean(C)-mean(C0)]) were measured on the day the first animal was removed due to large tumor size. For efficacy, the mean ΔT/C was based on the difference in tumor volume, for tolerability, on the fold-change in mean body weight. Mice with a single measure of 20% BWL were culled. One drug-related animal death occurred in the 24 h break combination group (day 8). Regressions represent a shrinkage of the tumor below the starting tumor size. *Regressions were observed in 1/8 mice and data from a single animal is presented.

| **Carboplatin** | **BAL0891** | **Efficacy**  **ΔT/C (day 23)** | **Tolerability**  **ΔT/C (day 23)** | **Regressions (%) (end of treatment day)** | **Tumor-free (%) (end of observation period)** |
| --- | --- | --- | --- | --- | --- |
| **-** | **7 mg/kg, (4h) 2QW, IV** | 0.64 | 0.94 |  |  |
| **-** | **7 mg/kg, (4h) QW, IV** | 0.69 | 0.98 |  |  |
| **60 mg/kg, QW, IV** | **-** | 0.72 | 0.97 |  |  |
| **60 mg/kg, QW, IV** | **7 mg/kg, (1h) 2QW, IV** | 0.21 | 0.94 | 20 (27)* | 12.5 |
| **60 mg/kg, QW, IV** | **7 mg/kg, (4h) 2QW, IV** | 0.20 | 0.96 | 4 (27)* | 12.5 |
| **60 mg/kg, QW, IV** | **7 mg/kg, (24h) 2QW, IV** | 0.32 | 0.95 |  |  |
| **60 mg/kg, QW, IV** | **7 mg/kg, (4h) QW, IV** | 0.46 | 0.97 |  |  |

**Supplementary Table 6. Cell culture conditions of cell lines used in proliferation assays.**

Cells were cultured in the indicated medium and grown at 37°C in 5% CO_2_. All cell lines were tested regularly to exclude mycoplasma infection.

| **Cell line** | **Company** | **Cat #** | **Lot** | **Medium** |
| --- | --- | --- | --- | --- |
| 769-P | ATCC | CRL-1933 |  | RPMI-1640, 10% (v/v) FCS, 1% (v/v) Penicillin/Streptomycin |
| 786-O | ATCC | CRL-1932 |  | RPMI-1640, 10% (v/v) FCS, 1% (v/v) Penicillin/Streptomycin |
| 233132/87 | DSMZ | ACC 201 | Lot 9 | RPMI-1640, 10% (v/v) FCS, 1% (v/v) Penicillin/Streptomycin |
| ACHN | ATCC | CRL-1611 |  | DMEM, 10% (v/v) FCS, 1% (v/v) Penicillin/Streptomycin |
| AGS | Sigma/ECACC | 89090402 | Lot 12H012 | Ham’s F12, 10% (v/v) FCS, 1% (v/v) Penicillin/Streptomycin, 2 mM Glutamine |
| AN3 CA | ATCC | HTB-111 |  | DMEM, 10% (v/v) FCS, 1% (v/v) Penicillin/Streptomycin |
| BC-3C | DSMZ | ACC 450 | Lot 2 | McCoy's 5A, 10% (v/v) FCS, 1% (v/v) Penicillin/Streptomycin |
| BT474 | ATCC | HTB-20 |  | Ham’s F12, 10% (v/v) FCS, 1% (v/v) Penicillin/Streptomycin, 2 mM Glutamine |
| BT549 | ATCC | HTB-122 | Lot 57741121 | RPMI-1640, 10% (v/v) FCS, 1% (v/v) Penicillin/Streptomycin, 2 mM L-Glutamine, 0.023 U/mL Insulin |
| Caki-1 | ATCC | HTB-46 |  | McCoy's 5A, 10% (v/v) FCS, 1% (v/v) Penicillin/Streptomycin |
| Caki-2 | ATCC | HTB-47 |  | McCoy's 5A, 10% (v/v) FCS, 1% (v/v) Penicillin/Streptomycin |
| CAL-120 | DSMZ | ACC459 | Lot 9 | DMEM, 10% (v/v) FCS, 1% (v/v) Penicillin/Streptomycin |
| CAL-29 | DSMZ | ACC515 | Lot 4 | DMEM, 10% (v/v) FCS, 1% (v/v) Penicillin/Streptomycin |
| CAL-51 | DSMZ | ACC302 | Lot 13 | DMEM, 10% (v/v) FCS, 1% (v/v) Penicillin/Streptomycin |
| CAL-54 | DSMZ | ACC 365 | Lot 5 | DMEM, 15% (v/v) FCS, 1% (v/v) Penicillin/Streptomycin, 4 mM L-glutamine, 1 mM sodium pyruvate, 0.4 µg/mL hydrocortisone, 10 ng/mL EGF |
| CAMA-1 | ATCC | HTB-21 | Lot 62486985 | DMEM, 10% (v/v) FCS, 1% (v/v) Penicillin/Streptomycin |
| DLD1 | Sigma | CLL0001-1VL |  | RPMI-1640, 10% (v/v) FCS, 1% (v/v) Penicillin/Streptomycin, 2 mM L-Glutamine, 1 mM Sodium pyruvate |
| Fu97 | JCRB | JCRB1074 | Lot 03132015 | DMEM, 10% (v/v) FCS, 1% (v/v) Penicillin/Streptomycin, 0.023 U/mL Insulin |
| G401 | Sigma | CLL0004-1VL |  | McCoy's 5A, 10% (v/v) FCS, 1% (v/v) Penicillin/Streptomycin |
| HCC1395 | ATCC | CRL-2324 | Lot 64146724 | DMEM, 10% (v/v) FCS, 1% (v/v) Penicillin/Streptomycin |
| HCC1500 | ATCC | CRL-2329 |  | RPMI-1640, 10% (v/v) FCS, 1% (v/v) Penicillin/Streptomycin |
| HCC1937 | ATCC | CRL-2336 | Lot 4714380 | RPMI-1640, 10% (v/v) FCS, 1% (v/v) Penicillin/Streptomycin, 2 mM L-Glutamine, 1% (v/v) MEM non-essential amino acids, 1 mM Sodium pyruvate |
| HCC1954 | ATCC | CRL-2338 | Lot 63681081 | RPMI-1640, 10% (v/v) FCS, 1% (v/v) Penicillin/Streptomycin, 1 mM Sodium pyruvate, 1% (v/v) Hepes |
| HCC70 | ATCC | CRL-2315 | Lot 61978349 | RPMI-1640, 10% (v/v) FCS, 1% (v/v) Penicillin/Streptomycin, 1 mM Sodium pyruvate, 1% (v/v) Hepes |
| HCT116 | ATCC | CCl-247 | Lot 5056139 | McCoy's 5A, 10% (v/v) FCS, 1% (v/v) Penicillin/Streptomycin |
| HCT-15 | ATCC | CCL-225 | Lot 710626977 | RPMI-1640, 10% (v/v) FCS, 1% (v/v) Penicillin/Streptomycin |
| HEC-1-A | ATCC | HTB-112 |  | McCoy's 5A, 10% (v/v) FCS, 1% (v/v) Penicillin/Streptomycin |
| HEC-1-B | ATCC | HTB-113 |  | DMEM, 10% (v/v) FCS, 1% (v/v) Penicillin/Streptomycin |
| HEC-251 | JCRB | JCRB1141 |  | DMEM, 15% (v/v) FCS, 1% (v/v) Penicillin/Streptomycin |
| HEC-265 | JCRB | JCRB1142 |  | DMEM, 15% (v/v) FCS, 1% (v/v) Penicillin/Streptomycin |
| HEC-50B | JCRB | JCRB1145 | Lot 10182013 | DMEM, 15% (v/v) FCS, 1% (v/v) Penicillin/Streptomycin |
| HEC-59 | Accegen | ABC-TC0348 | Lot 09042012 | DMEM, 10% (v/v) FCS, 1% (v/v) Penicillin/Streptomycin |
| HEC-6 | JCRB | JCRB1118 |  | DMEM, 15% (v/v) FCS, 1% (v/v) Penicillin/Streptomycin |
| HGC-27 | ECACC | 94042256 | Lot 17H077 | MEM, 10% (v/v) FCS, 1% (v/v) Penicillin/Streptomycin |
| HMEC | Lonza | CC-2551 | Lot 7F3285 | MEBM, SingleQuots growth factors |
| HRT-18 | ECACC | 86040306 | Lot 12D002 | RPMI-1640, 10% (v/v) FCS, 1% (v/v) Penicillin/Streptomycin, 2 mM L-Glutamine |
| Hs578T | ATCC | HTB-126 | Lot 63087041 | DMEM, 10% (v/v) FCS, 1% (v/v) Penicillin/Streptomycin |
| HS68 | ATCC | CRL-1635 | Lot 4748910 | DMEM, 10% (v/v) FCS, 1% (v/v) Penicillin/Streptomycin, 1 mM Sodium pyruvate |
| Hs746T | ATCC | HTB-135 | Lot 70021638 | DMEM, 10% (v/v) FCS, 1% (v/v) Penicillin/Streptomycin |
| HT1197 | ECACC | 87032403 |  | DMEM, 10% (v/v) FCS, 1% (v/v) Penicillin/Streptomycin, 2 mM L-Glutamine, 1% (v/v) MEM NEAA |
| HT1376 | ECACC | 87032402 |  | DMEM, 10% (v/v) FCS, 1% (v/v) Penicillin/Streptomycin, 2 mM L-Glutamine, 1% (v/v) MEM NEAA |
| HT-29 | ATCC | HTB-38 |  | RPMI-1640 10% (v/v) FCS, 1% (v/v) Penicillin/Streptomycin, 2 mM L-Glutamine, 1% (v/v) MEM NEAA |
| HT55 | ECACC | 85061105 |  | RPMI-1640 10% (v/v) FCS, 1% (v/v) Penicillin/Streptomycin, 2 mM L-Glutamine, 1% (v/v) MEM NEAA |
| IM95 | JCRB | JCRB1075 | Lot 09302016 | DMEM, 10% (v/v) FCS, 1% (v/v) Penicillin/Streptomycin, 0.023 U/mL Insulin |
| J-82 | ATCC | HTB-1 |  | DMEM, 10% (v/v) FCS, 1% (v/v) Penicillin/Streptomycin, 1 mM Sodium pyruvate |
| JMSU1 | DSMZ | ACC 505 | Lot 3 | RPMI-1640, 10% (v/v) FCS, 1% (v/v) Penicillin/Streptomycin, 2 mM L-Glutamine |
| Kato-III | Sigma/ECACC | 86093004 | Lot 13G015 | RPMI-1640, 20% (v/v) FCS, 1% (v/v) Penicillin/Streptomycin, 2 mM L-Glutamine |
| KU-19-19 | DSMZ | ACC 395 | Lot 5 | RPMI-1640, 10% (v/v) FCS, 1% (v/v) Penicillin/Streptomycin |
| LoVo | ATCC | CCL-229 | Lot 4031215 | RPMI-1640, 10% (v/v) FCS, 1% (v/v) Penicillin/Streptomycin, 2 mM L-Glutamine, 1% (v/v) MEM non-essential amino acids, 1 mM Sodium pyruvate |
| LS174T | ATCC | CL-188 | Lot 57624482 | DMEM, 10% (v/v) FCS, 1% (v/v) Penicillin/Streptomycin, 1 mM Sodium pyruvate |
| MCF7 | ATCC | HTB-22 | Lot 7529960 | DMEM, 10% (v/v) FCS, 1% (v/v) Penicillin/Streptomycin, 1 mM Sodium pyruvate, 1% (v/v) MEM NEAA |
| MDA-MB-231 | ATCC | HTB-26 | Lot 5069789 | DMEM, 10% (v/v) FCS, 1% (v/v) Penicillin/Streptomycin, 1 mM Sodium pyruvate |
| MDA-MB-453 | ATCC | HTB-131 | Lot 4019064 | DMEM, 10% (v/v) FCS, 1% (v/v) Penicillin/Streptomycin, 1 mM Sodium pyruvate |
| MDA-MB-468 | ATCC | HTB-132 | Lot 57675411 | DMEM, 10% (v/v) FCS, 1% (v/v) Penicillin/Streptomycin, 1 mM Sodium pyruvate |
| MDST8 | ECACC | 99011801 | Lot 13K001 | DMEM, 10% (v/v) FCS, 1% (v/v) Penicillin/Streptomycin, 2 mM L-Glutamine |
| MFE-280 | ECACC | 98050131 | Lot 11J030 | DMEM, 10% (v/v) FCS, 1% (v/v) Penicillin/Streptomycin, 2 mM L-Glutamine |
| MFE-296 | ECACC | 98031101 | Lot 12F039 | DMEM, 10% (v/v) FCS, 1% (v/v) Penicillin/Streptomycin, 2 mM L-Glutamine |
| NCI-H716 | ATCC | CCL-251 | Lot 63990056 | RPMI-1640, 10% (v/v) FCS, 1% (v/v) Penicillin/Streptomycin |
| NCI-N87 | ATCC | CRL-5822 | Lot 70014503 | RPMI-1640, 10% (v/v) FCS, 1% (v/v) Penicillin/Streptomycin |
| NUGC-3 | JCRB | JCRB0822 | Lot 11162017 | RPMI-1640, 10% (v/v) FCS, 1% (v/v) Penicillin/Streptomycin |
| OCUM-1 | JCRB | JCRB0192 |  | DMEM, 10% (v/v) FCS, 1% (v/v) Penicillin/Streptomycin, 1 mM Sodium pyruvate |
| RCC4 vector | Sigma | 3112702 |  | DMEM, 10% (v/v) FCS, 1% (v/v) Penicillin/Streptomycin, 2 mM L-Glutamine |
| RERF-GC-1B | JCRB | JCRB1009 | Lot 07192016 | RPMI-1640, 10% (v/v) FCS, 1% (v/v) Penicillin/Streptomycin |
| RKO | ATCC | CRL-2577 | Lot 62300117 | DMEM, 10% (v/v) FCS, 1% (v/v) Penicillin/Streptomycin |
| RL95-2 | ATCC | CRL-1671 | Lot 62130010 | DMEM, 10% (v/v) FCS, 1% (v/v) Penicillin/Streptomycin |
| RT112/84 | ECACC | 85061106 |  | DMEM, 10% (v/v) FCS, 1% (v/v) Penicillin/Streptomycin, 2 mM L-Glutamine, 1% (v/v) MEM NEAA |
| RT4 | ECACC | 91091914 |  | McCoy's 5A, 10% (v/v) FCS, 1% (v/v) Penicillin/Streptomycin, 2 mM Glutamine |
| SKBR3 | ATCC | HTB-30 |  | McCoy's 5A, 10% (v/v) FCS, 1% (v/v) Penicillin/Streptomycin |
| SNG-M | JCRB | JCRB0179 | Lot 07212009 | Ham’s F12, 10% (v/v) FCS, 1% (v/v) Penicillin/Streptomycin, 2 mM Glutamine |
| SNU16 | ATCC | CRL-5974 |  | DMEM, 10% (v/v) FCS, 1% (v/v) Penicillin/Streptomycin |
| SNU-349 | KCLB | 349 |  | RPMI-1640, 10% (v/v) FCS, 1% (v/v) Penicillin/Streptomycin |
| SW-1710 | DSMZ | ACC 426 | Lot 3 | DMEM, 15% (v/v) FCS, 1% (v/v) Penicillin/Streptomycin |
| SW403 | DSMZ | ACC 294 | Lot 4 | DMEM, 10% (v/v) FCS, 1% (v/v) Penicillin/Streptomycin |
| SW48 | ATCC | CCL-231 | Lot 3247840 | DMEM, 10% (v/v) FCS, 1% (v/v) Penicillin/Streptomycin, 1 mM Sodium pyruvate |
| SW480 | ATCC | CCL-228 | Lot 4487738 | RPMI-1640, 10% (v/v) FCS, 1% (v/v) Penicillin/Streptomycin, 2 mM L-Glutamine, 1% (v/v) MEM non-essential amino acids, 1 mM Sodium pyruvate |
| SW620 | ATCC | CCL-227 | Lot 58483168 | RPMI-1640 10% (v/v) FCS, 1% (v/v) Penicillin/Streptomycin, 2 mM L-Glutamine, 1% (v/v) MEM NEAA |
| SW-780 | ATCC | CRL-2169 | Lot 61978343 | DMEM, 10% (v/v) FCS, 1% (v/v) Penicillin/Streptomycin |
| SW948 | ATCC | CCL-237 | Lot 62522213 | RPMI-1640, 10% (v/v) FCS, 1% (v/v) Penicillin/Streptomycin |
| T24 | DSMZ | ACC 376 | Lot 5 | DMEM, 10% (v/v) FCS, 1% (v/v) Penicillin/Streptomycin |
| T47D | ATCC | HTB-133 |  | DMEM, 10% (v/v) FCS, 1% (v/v) Penicillin/Streptomycin, 1 mM Sodium pyruvate |
| T84 | ECACC | 88021101 |  | Ham’s F12, 10% (v/v) FCS, 1% (v/v) Penicillin/Streptomycin, 2 mM Glutamine |
| TCC-SUP | DSMZ | ACC 377 | Lot 3 | DMEM, 15% (v/v) FCS, 1% (v/v) Penicillin/Streptomycin |
| ZR-75-1 | ATCC | CRL-1504 |  | RPMI-1640, 10% (v/v) FCS, 1% (v/v) Penicillin/Streptomycin |
