# Supplementary material for: Dual TTK/PLK1 inhibition has potent anticancer activity in TNBC as monotherapy and in combination
Source: Front Oncol. 2024 Aug 9;14:1447807. doi: 10.3389/fonc.2024.1447807 (PMC11341980; doi:10.3389/fonc.2024.1447807)
Supplement: Supplementary file 2 [file Presentation_1.pptx]

## Slide 1
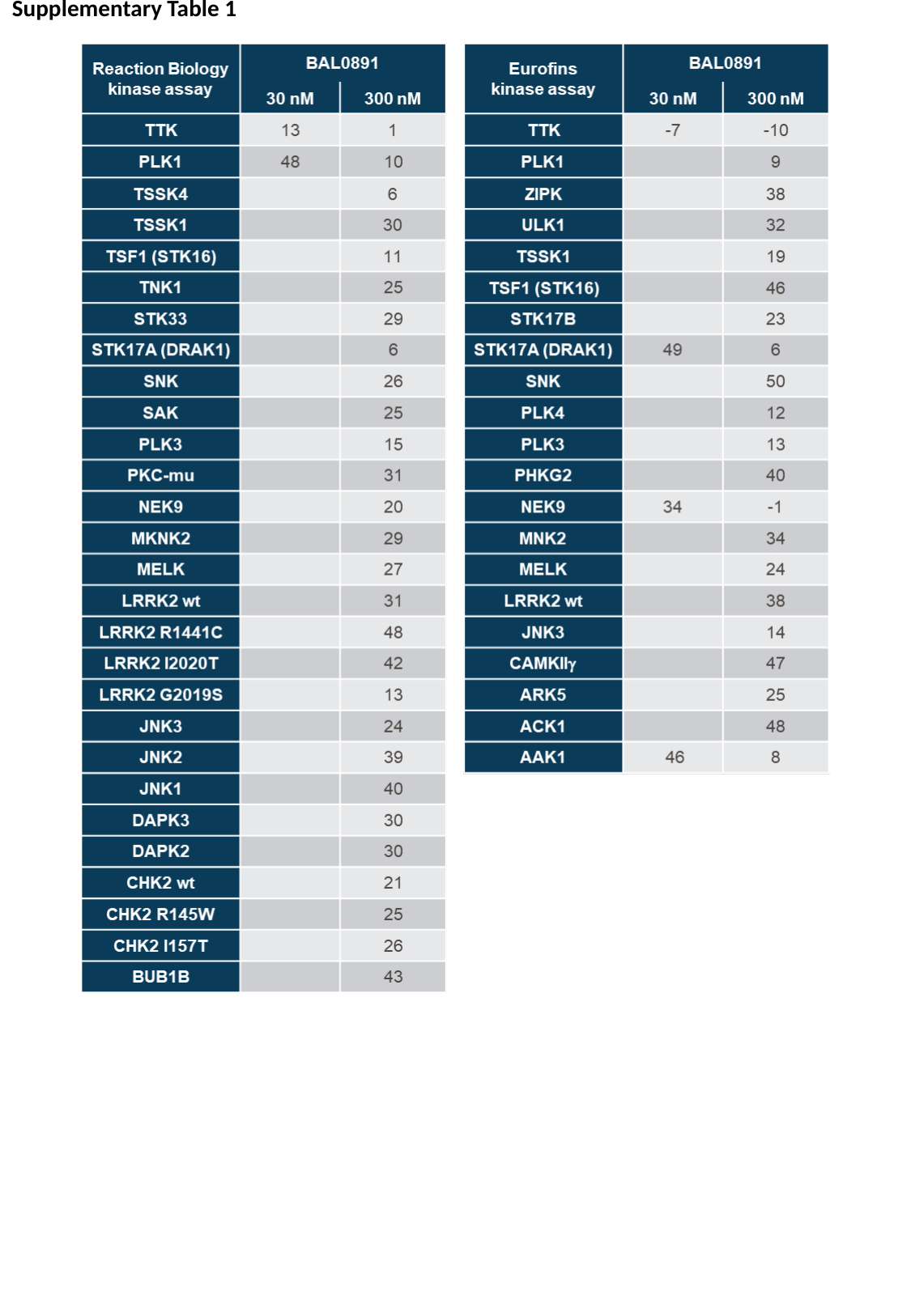

Supplementary Table 1

## Slide 2
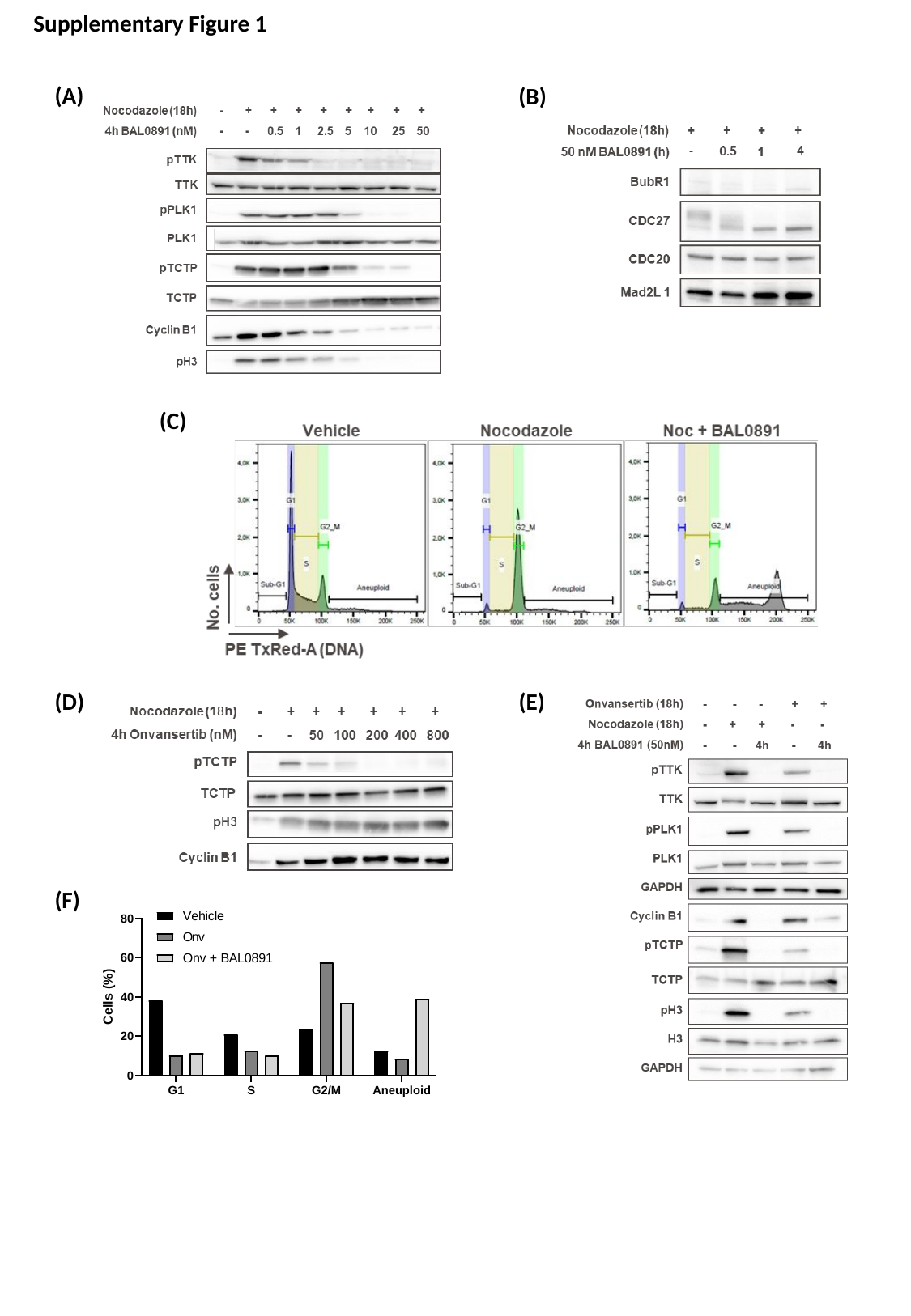

Supplementary Figure 1
(A)
(B)
(C)
(D)
(E)
(F)

## Slide 3
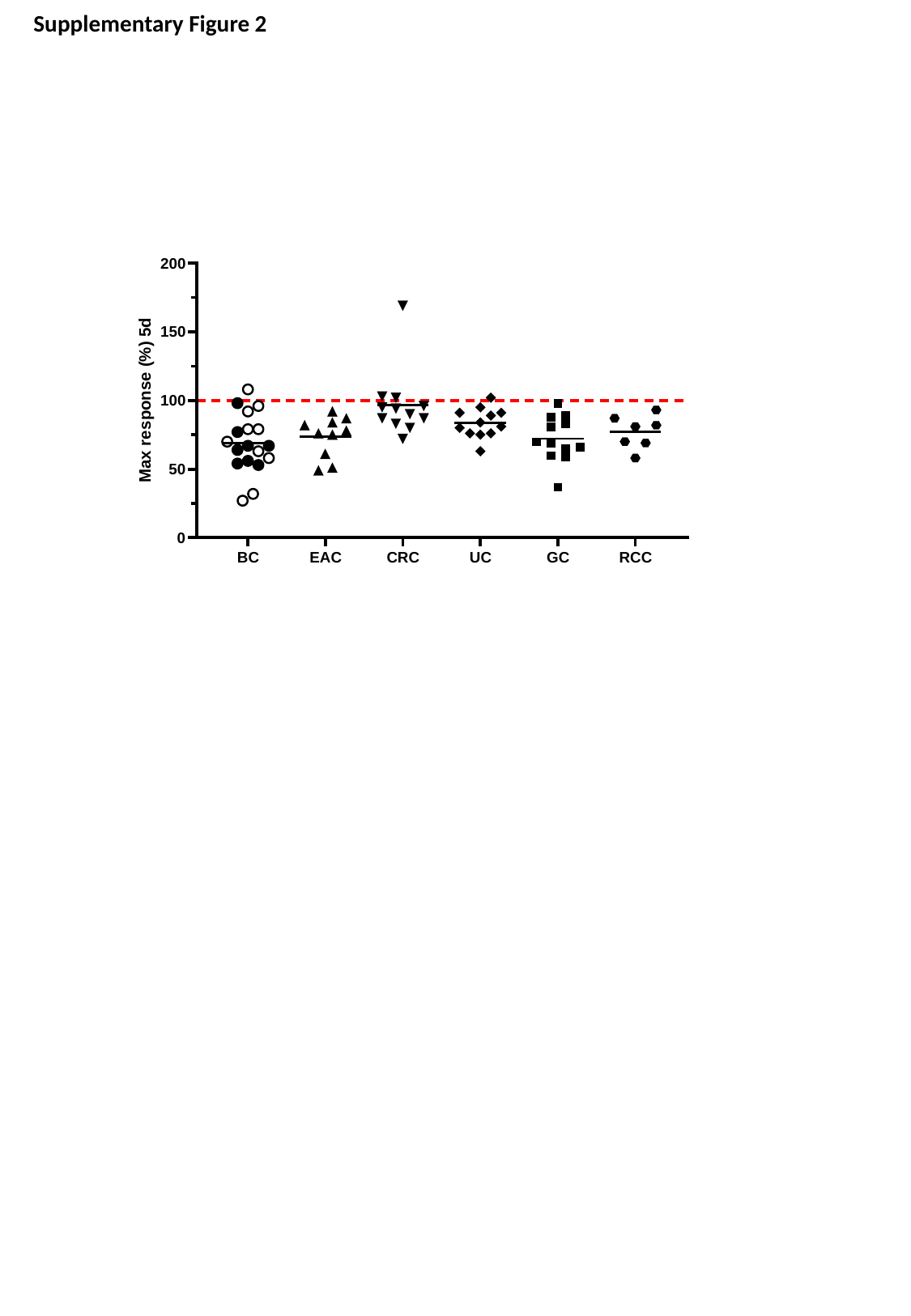

Supplementary Figure 2

## Slide 4
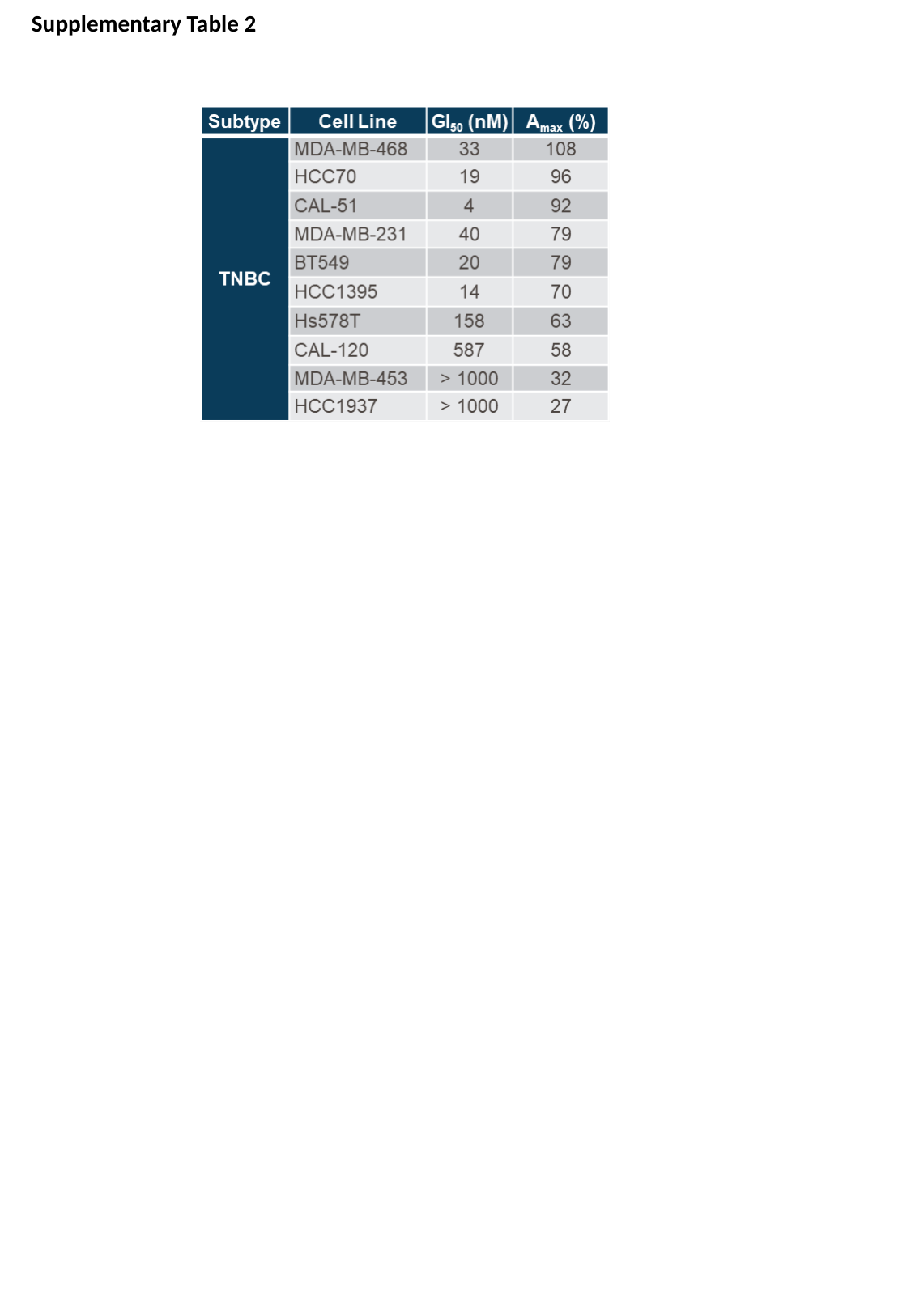

Supplementary Table 2

## Slide 5
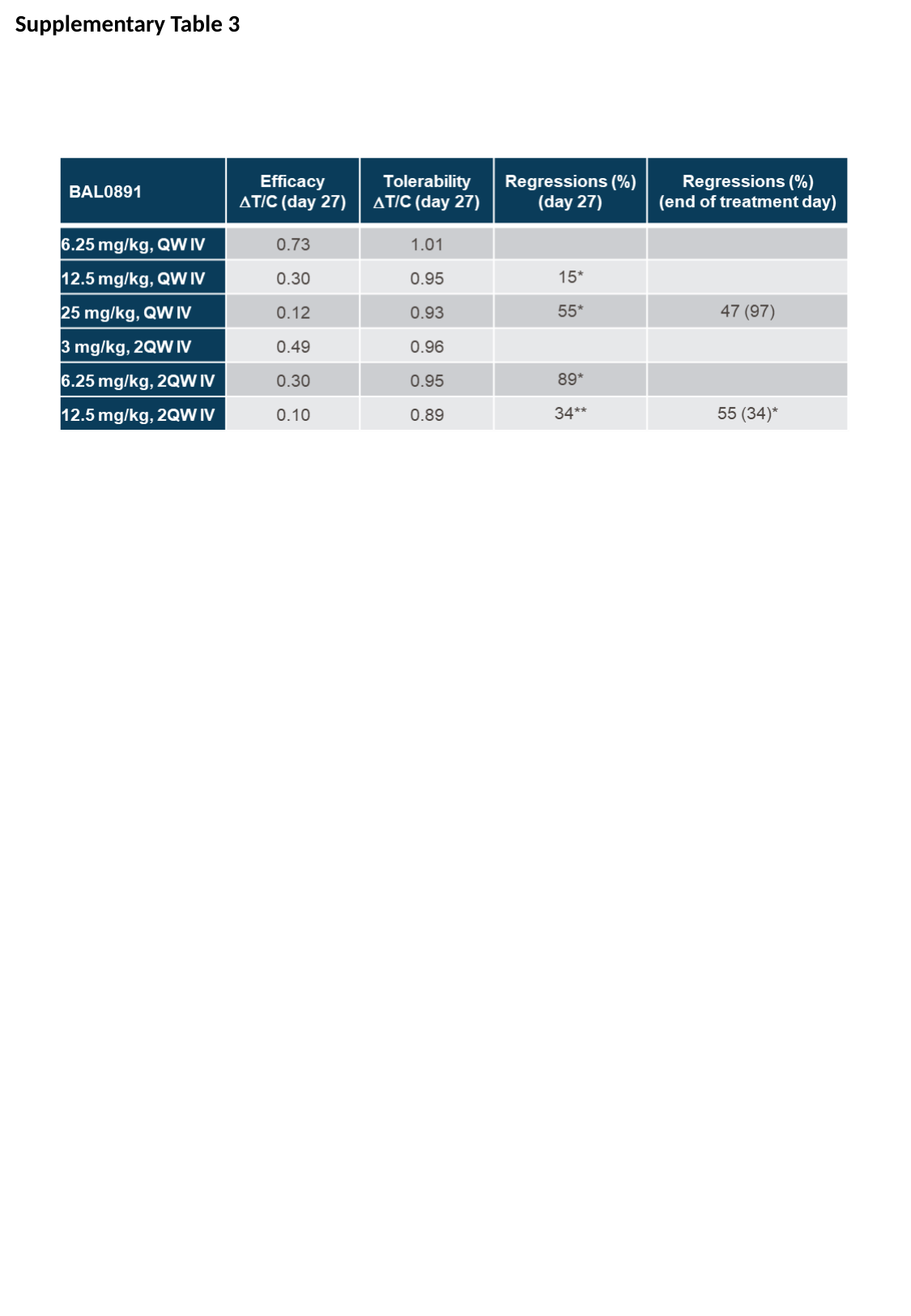

Supplementary Table 3

## Slide 6
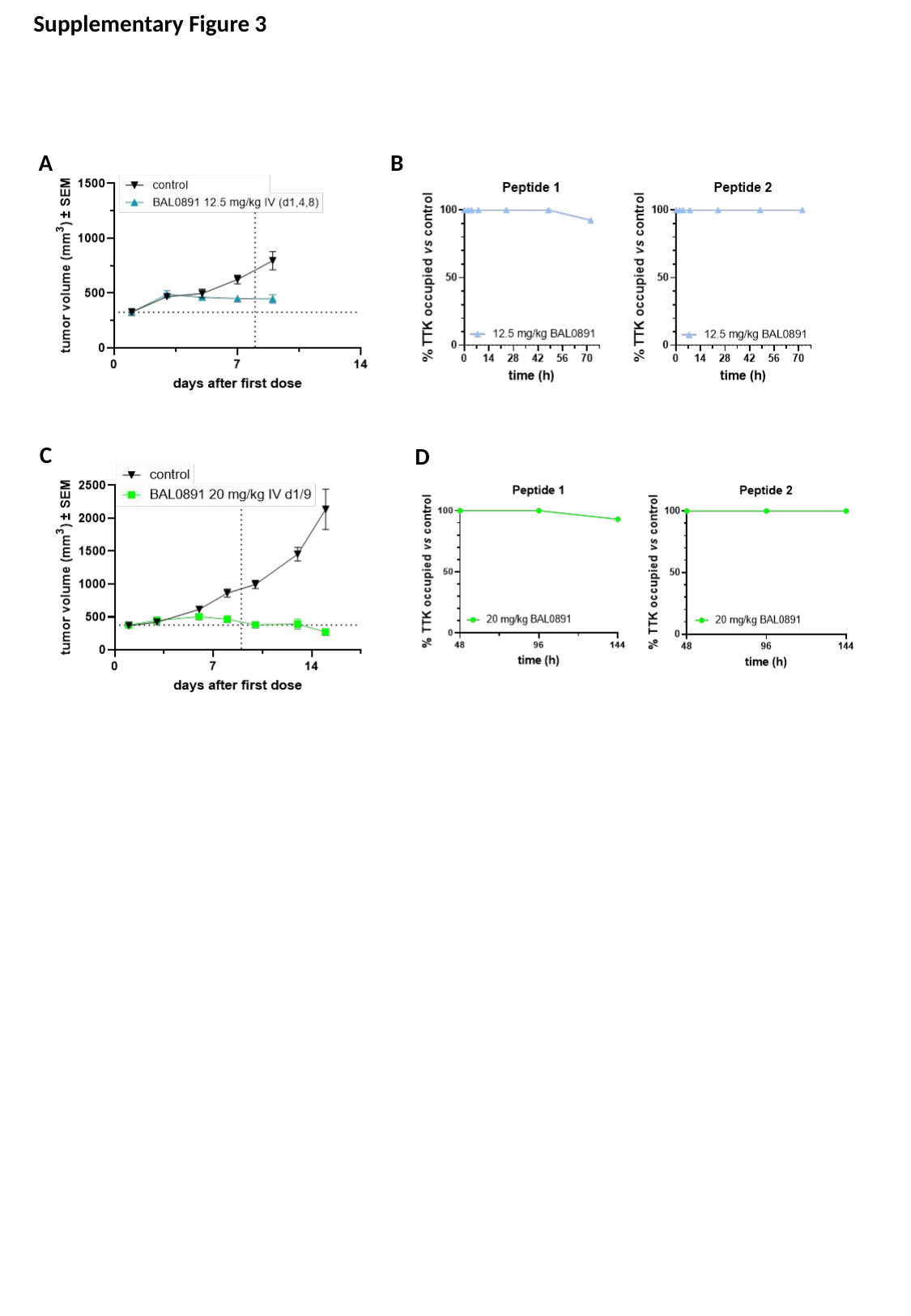

Supplementary Figure 3
A
B
C
D

## Slide 7
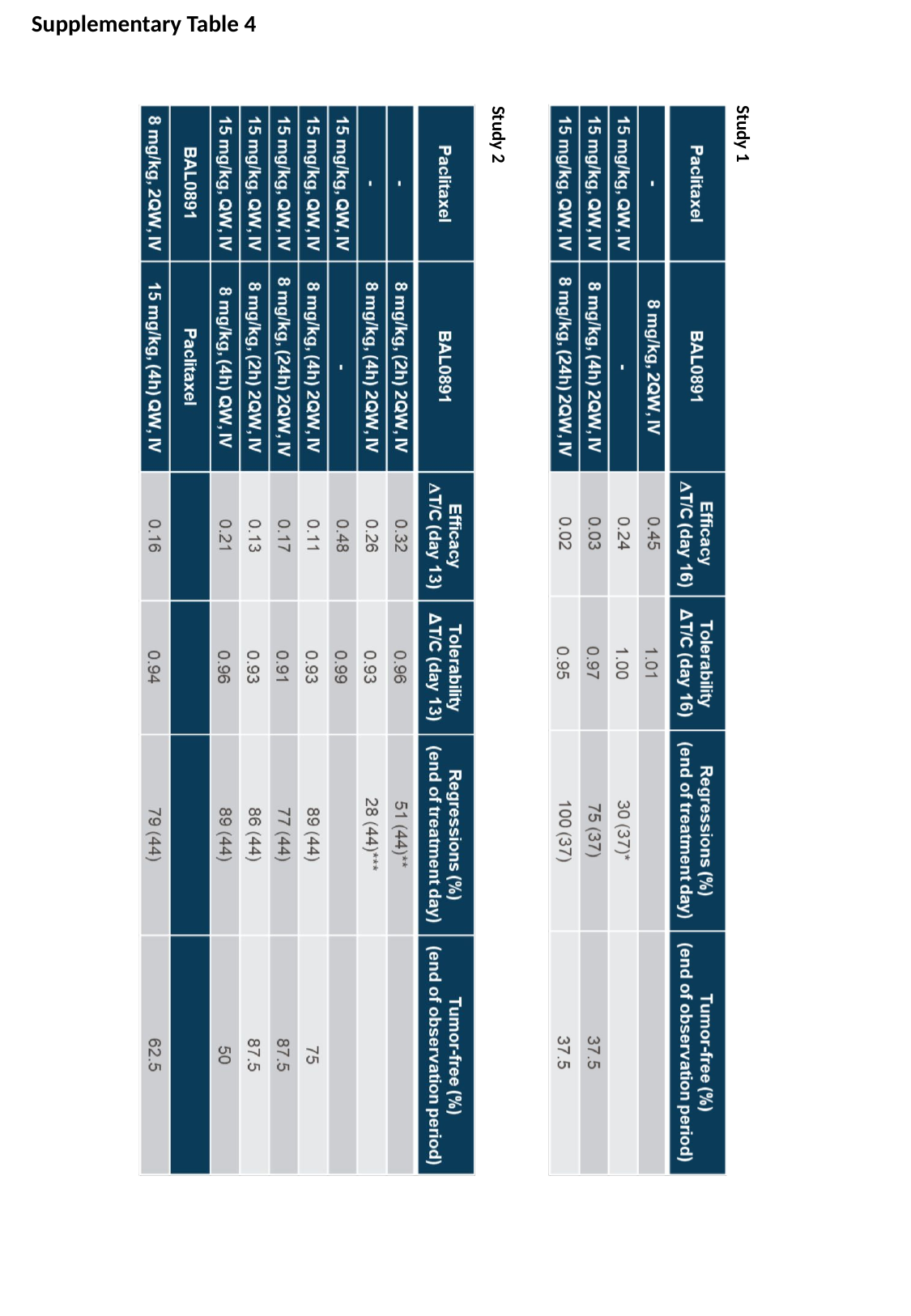

Supplementary Table 4
Study 1
Study 2

## Slide 8
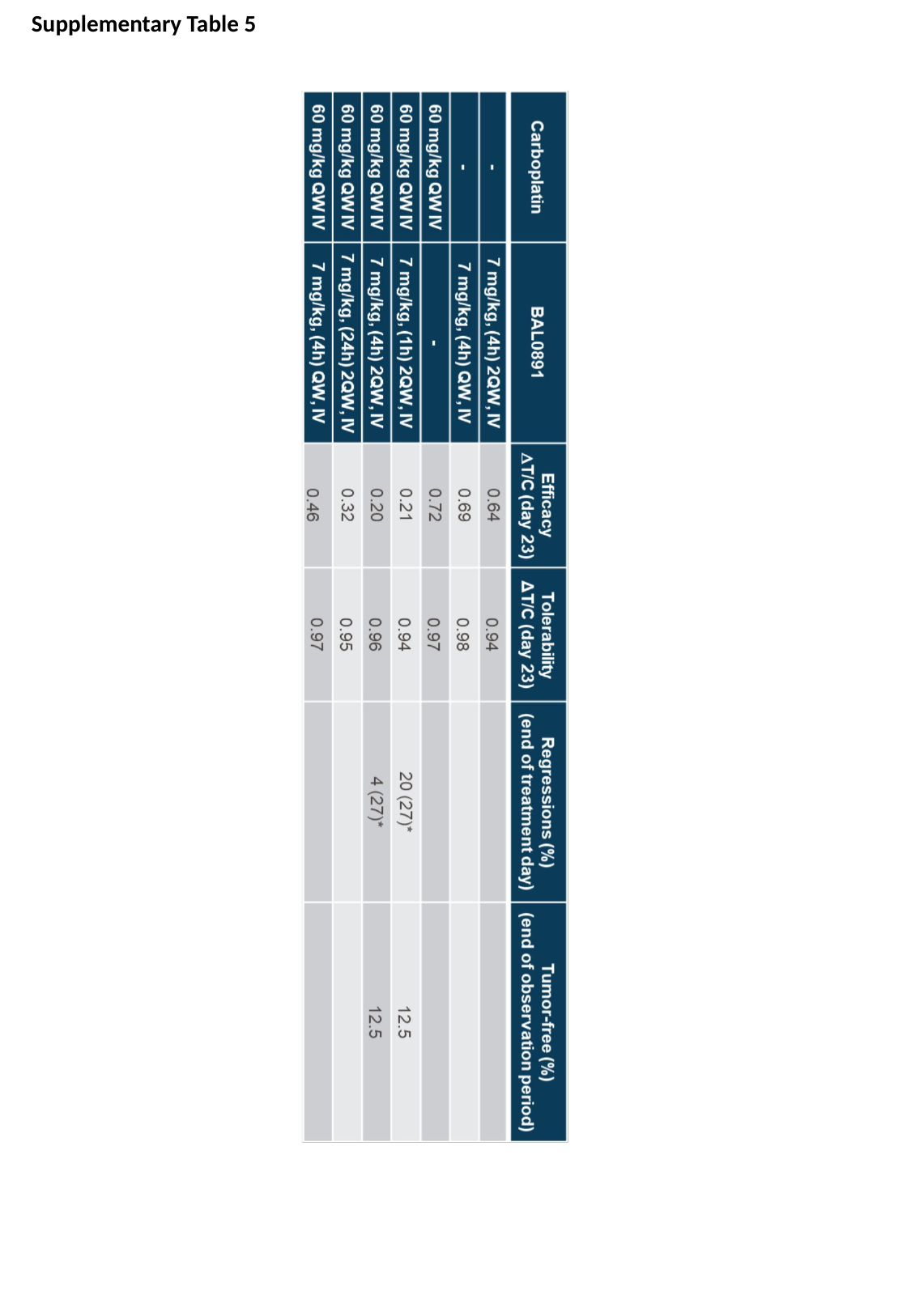

Supplementary Table 5

## Slide 9
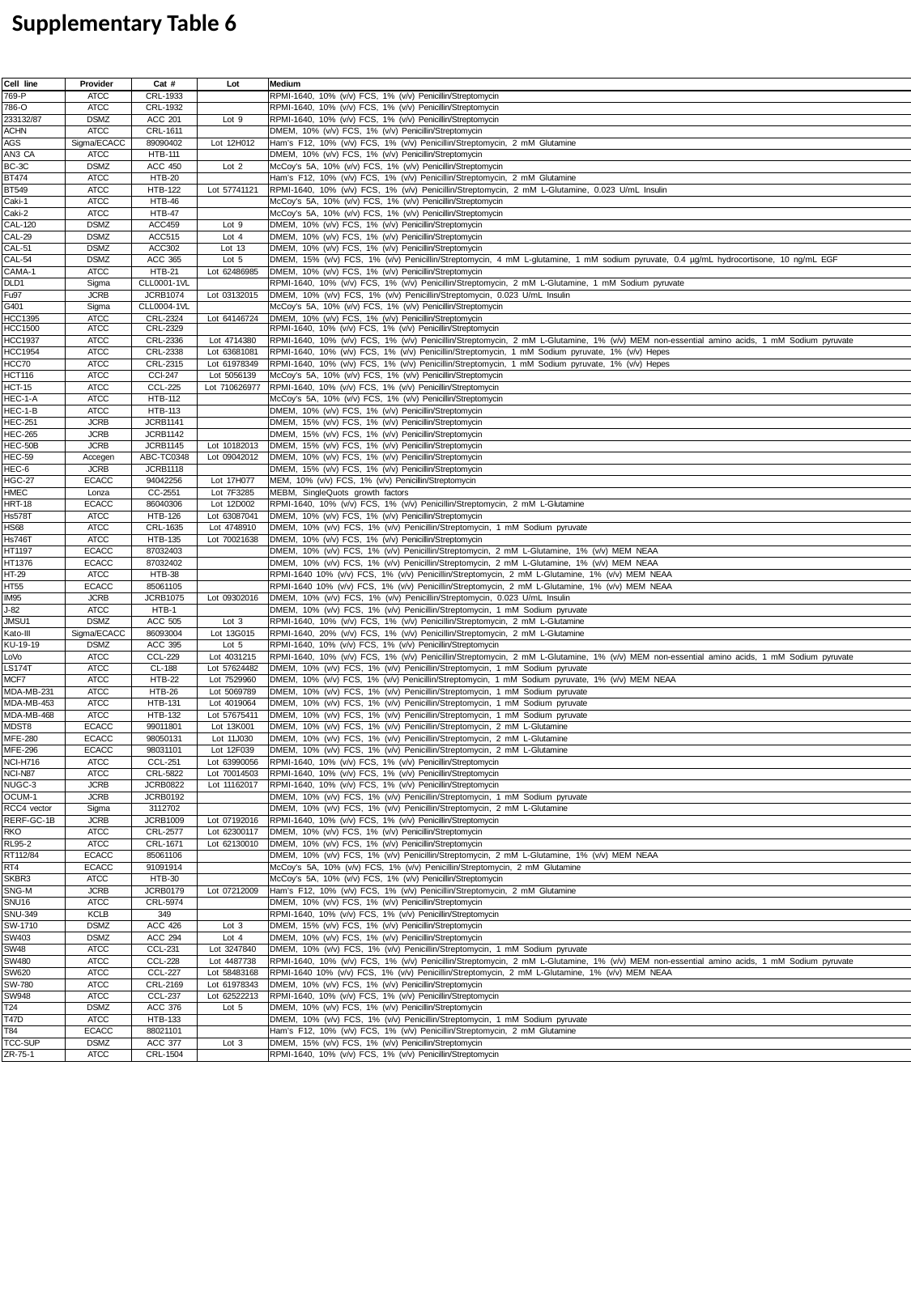

Supplementary Table 6
| Cell line | Provider | Cat # | Lot | Medium |
| --- | --- | --- | --- | --- |
| 769-P | ATCC | CRL-1933 | | RPMI-1640, 10% (v/v) FCS, 1% (v/v) Penicillin/Streptomycin |
| 786-O | ATCC | CRL-1932 | | RPMI-1640, 10% (v/v) FCS, 1% (v/v) Penicillin/Streptomycin |
| 233132/87 | DSMZ | ACC 201 | Lot 9 | RPMI-1640, 10% (v/v) FCS, 1% (v/v) Penicillin/Streptomycin |
| ACHN | ATCC | CRL-1611 | | DMEM, 10% (v/v) FCS, 1% (v/v) Penicillin/Streptomycin |
| AGS | Sigma/ECACC | 89090402 | Lot 12H012 | Ham’s F12, 10% (v/v) FCS, 1% (v/v) Penicillin/Streptomycin, 2 mM Glutamine |
| AN3 CA | ATCC | HTB-111 | | DMEM, 10% (v/v) FCS, 1% (v/v) Penicillin/Streptomycin |
| BC-3C | DSMZ | ACC 450 | Lot 2 | McCoy's 5A, 10% (v/v) FCS, 1% (v/v) Penicillin/Streptomycin |
| BT474 | ATCC | HTB-20 | | Ham’s F12, 10% (v/v) FCS, 1% (v/v) Penicillin/Streptomycin, 2 mM Glutamine |
| BT549 | ATCC | HTB-122 | Lot 57741121 | RPMI-1640, 10% (v/v) FCS, 1% (v/v) Penicillin/Streptomycin, 2 mM L-Glutamine, 0.023 U/mL Insulin |
| Caki-1 | ATCC | HTB-46 | | McCoy's 5A, 10% (v/v) FCS, 1% (v/v) Penicillin/Streptomycin |
| Caki-2 | ATCC | HTB-47 | | McCoy's 5A, 10% (v/v) FCS, 1% (v/v) Penicillin/Streptomycin |
| CAL-120 | DSMZ | ACC459 | Lot 9 | DMEM, 10% (v/v) FCS, 1% (v/v) Penicillin/Streptomycin |
| CAL-29 | DSMZ | ACC515 | Lot 4 | DMEM, 10% (v/v) FCS, 1% (v/v) Penicillin/Streptomycin |
| CAL-51 | DSMZ | ACC302 | Lot 13 | DMEM, 10% (v/v) FCS, 1% (v/v) Penicillin/Streptomycin |
| CAL-54 | DSMZ | ACC 365 | Lot 5 | DMEM, 15% (v/v) FCS, 1% (v/v) Penicillin/Streptomycin, 4 mM L-glutamine, 1 mM sodium pyruvate, 0.4 µg/mL hydrocortisone, 10 ng/mL EGF |
| CAMA-1 | ATCC | HTB-21 | Lot 62486985 | DMEM, 10% (v/v) FCS, 1% (v/v) Penicillin/Streptomycin |
| DLD1 | Sigma | CLL0001-1VL | | RPMI-1640, 10% (v/v) FCS, 1% (v/v) Penicillin/Streptomycin, 2 mM L-Glutamine, 1 mM Sodium pyruvate |
| Fu97 | JCRB | JCRB1074 | Lot 03132015 | DMEM, 10% (v/v) FCS, 1% (v/v) Penicillin/Streptomycin, 0.023 U/mL Insulin |
| G401 | Sigma | CLL0004-1VL | | McCoy's 5A, 10% (v/v) FCS, 1% (v/v) Penicillin/Streptomycin |
| HCC1395 | ATCC | CRL-2324 | Lot 64146724 | DMEM, 10% (v/v) FCS, 1% (v/v) Penicillin/Streptomycin |
| HCC1500 | ATCC | CRL-2329 | | RPMI-1640, 10% (v/v) FCS, 1% (v/v) Penicillin/Streptomycin |
| HCC1937 | ATCC | CRL-2336 | Lot 4714380 | RPMI-1640, 10% (v/v) FCS, 1% (v/v) Penicillin/Streptomycin, 2 mM L-Glutamine, 1% (v/v) MEM non-essential amino acids, 1 mM Sodium pyruvate |
| HCC1954 | ATCC | CRL-2338 | Lot 63681081 | RPMI-1640, 10% (v/v) FCS, 1% (v/v) Penicillin/Streptomycin, 1 mM Sodium pyruvate, 1% (v/v) Hepes |
| HCC70 | ATCC | CRL-2315 | Lot 61978349 | RPMI-1640, 10% (v/v) FCS, 1% (v/v) Penicillin/Streptomycin, 1 mM Sodium pyruvate, 1% (v/v) Hepes |
| HCT116 | ATCC | CCl-247 | Lot 5056139 | McCoy's 5A, 10% (v/v) FCS, 1% (v/v) Penicillin/Streptomycin |
| HCT-15 | ATCC | CCL-225 | Lot 710626977 | RPMI-1640, 10% (v/v) FCS, 1% (v/v) Penicillin/Streptomycin |
| HEC-1-A | ATCC | HTB-112 | | McCoy's 5A, 10% (v/v) FCS, 1% (v/v) Penicillin/Streptomycin |
| HEC-1-B | ATCC | HTB-113 | | DMEM, 10% (v/v) FCS, 1% (v/v) Penicillin/Streptomycin |
| HEC-251 | JCRB | JCRB1141 | | DMEM, 15% (v/v) FCS, 1% (v/v) Penicillin/Streptomycin |
| HEC-265 | JCRB | JCRB1142 | | DMEM, 15% (v/v) FCS, 1% (v/v) Penicillin/Streptomycin |
| HEC-50B | JCRB | JCRB1145 | Lot 10182013 | DMEM, 15% (v/v) FCS, 1% (v/v) Penicillin/Streptomycin |
| HEC-59 | Accegen | ABC-TC0348 | Lot 09042012 | DMEM, 10% (v/v) FCS, 1% (v/v) Penicillin/Streptomycin |
| HEC-6 | JCRB | JCRB1118 | | DMEM, 15% (v/v) FCS, 1% (v/v) Penicillin/Streptomycin |
| HGC-27 | ECACC | 94042256 | Lot 17H077 | MEM, 10% (v/v) FCS, 1% (v/v) Penicillin/Streptomycin |
| HMEC | Lonza | CC-2551 | Lot 7F3285 | MEBM, SingleQuots growth factors |
| HRT-18 | ECACC | 86040306 | Lot 12D002 | RPMI-1640, 10% (v/v) FCS, 1% (v/v) Penicillin/Streptomycin, 2 mM L-Glutamine |
| Hs578T | ATCC | HTB-126 | Lot 63087041 | DMEM, 10% (v/v) FCS, 1% (v/v) Penicillin/Streptomycin |
| HS68 | ATCC | CRL-1635 | Lot 4748910 | DMEM, 10% (v/v) FCS, 1% (v/v) Penicillin/Streptomycin, 1 mM Sodium pyruvate |
| Hs746T | ATCC | HTB-135 | Lot 70021638 | DMEM, 10% (v/v) FCS, 1% (v/v) Penicillin/Streptomycin |
| HT1197 | ECACC | 87032403 | | DMEM, 10% (v/v) FCS, 1% (v/v) Penicillin/Streptomycin, 2 mM L-Glutamine, 1% (v/v) MEM NEAA |
| HT1376 | ECACC | 87032402 | | DMEM, 10% (v/v) FCS, 1% (v/v) Penicillin/Streptomycin, 2 mM L-Glutamine, 1% (v/v) MEM NEAA |
| HT-29 | ATCC | HTB-38 | | RPMI-1640 10% (v/v) FCS, 1% (v/v) Penicillin/Streptomycin, 2 mM L-Glutamine, 1% (v/v) MEM NEAA |
| HT55 | ECACC | 85061105 | | RPMI-1640 10% (v/v) FCS, 1% (v/v) Penicillin/Streptomycin, 2 mM L-Glutamine, 1% (v/v) MEM NEAA |
| IM95 | JCRB | JCRB1075 | Lot 09302016 | DMEM, 10% (v/v) FCS, 1% (v/v) Penicillin/Streptomycin, 0.023 U/mL Insulin |
| J-82 | ATCC | HTB-1 | | DMEM, 10% (v/v) FCS, 1% (v/v) Penicillin/Streptomycin, 1 mM Sodium pyruvate |
| JMSU1 | DSMZ | ACC 505 | Lot 3 | RPMI-1640, 10% (v/v) FCS, 1% (v/v) Penicillin/Streptomycin, 2 mM L-Glutamine |
| Kato-III | Sigma/ECACC | 86093004 | Lot 13G015 | RPMI-1640, 20% (v/v) FCS, 1% (v/v) Penicillin/Streptomycin, 2 mM L-Glutamine |
| KU-19-19 | DSMZ | ACC 395 | Lot 5 | RPMI-1640, 10% (v/v) FCS, 1% (v/v) Penicillin/Streptomycin |
| LoVo | ATCC | CCL-229 | Lot 4031215 | RPMI-1640, 10% (v/v) FCS, 1% (v/v) Penicillin/Streptomycin, 2 mM L-Glutamine, 1% (v/v) MEM non-essential amino acids, 1 mM Sodium pyruvate |
| LS174T | ATCC | CL-188 | Lot 57624482 | DMEM, 10% (v/v) FCS, 1% (v/v) Penicillin/Streptomycin, 1 mM Sodium pyruvate |
| MCF7 | ATCC | HTB-22 | Lot 7529960 | DMEM, 10% (v/v) FCS, 1% (v/v) Penicillin/Streptomycin, 1 mM Sodium pyruvate, 1% (v/v) MEM NEAA |
| MDA-MB-231 | ATCC | HTB-26 | Lot 5069789 | DMEM, 10% (v/v) FCS, 1% (v/v) Penicillin/Streptomycin, 1 mM Sodium pyruvate |
| MDA-MB-453 | ATCC | HTB-131 | Lot 4019064 | DMEM, 10% (v/v) FCS, 1% (v/v) Penicillin/Streptomycin, 1 mM Sodium pyruvate |
| MDA-MB-468 | ATCC | HTB-132 | Lot 57675411 | DMEM, 10% (v/v) FCS, 1% (v/v) Penicillin/Streptomycin, 1 mM Sodium pyruvate |
| MDST8 | ECACC | 99011801 | Lot 13K001 | DMEM, 10% (v/v) FCS, 1% (v/v) Penicillin/Streptomycin, 2 mM L-Glutamine |
| MFE-280 | ECACC | 98050131 | Lot 11J030 | DMEM, 10% (v/v) FCS, 1% (v/v) Penicillin/Streptomycin, 2 mM L-Glutamine |
| MFE-296 | ECACC | 98031101 | Lot 12F039 | DMEM, 10% (v/v) FCS, 1% (v/v) Penicillin/Streptomycin, 2 mM L-Glutamine |
| NCI-H716 | ATCC | CCL-251 | Lot 63990056 | RPMI-1640, 10% (v/v) FCS, 1% (v/v) Penicillin/Streptomycin |
| NCI-N87 | ATCC | CRL-5822 | Lot 70014503 | RPMI-1640, 10% (v/v) FCS, 1% (v/v) Penicillin/Streptomycin |
| NUGC-3 | JCRB | JCRB0822 | Lot 11162017 | RPMI-1640, 10% (v/v) FCS, 1% (v/v) Penicillin/Streptomycin |
| OCUM-1 | JCRB | JCRB0192 | | DMEM, 10% (v/v) FCS, 1% (v/v) Penicillin/Streptomycin, 1 mM Sodium pyruvate |
| RCC4 vector | Sigma | 3112702 | | DMEM, 10% (v/v) FCS, 1% (v/v) Penicillin/Streptomycin, 2 mM L-Glutamine |
| RERF-GC-1B | JCRB | JCRB1009 | Lot 07192016 | RPMI-1640, 10% (v/v) FCS, 1% (v/v) Penicillin/Streptomycin |
| RKO | ATCC | CRL-2577 | Lot 62300117 | DMEM, 10% (v/v) FCS, 1% (v/v) Penicillin/Streptomycin |
| RL95-2 | ATCC | CRL-1671 | Lot 62130010 | DMEM, 10% (v/v) FCS, 1% (v/v) Penicillin/Streptomycin |
| RT112/84 | ECACC | 85061106 | | DMEM, 10% (v/v) FCS, 1% (v/v) Penicillin/Streptomycin, 2 mM L-Glutamine, 1% (v/v) MEM NEAA |
| RT4 | ECACC | 91091914 | | McCoy's 5A, 10% (v/v) FCS, 1% (v/v) Penicillin/Streptomycin, 2 mM Glutamine |
| SKBR3 | ATCC | HTB-30 | | McCoy's 5A, 10% (v/v) FCS, 1% (v/v) Penicillin/Streptomycin |
| SNG-M | JCRB | JCRB0179 | Lot 07212009 | Ham’s F12, 10% (v/v) FCS, 1% (v/v) Penicillin/Streptomycin, 2 mM Glutamine |
| SNU16 | ATCC | CRL-5974 | | DMEM, 10% (v/v) FCS, 1% (v/v) Penicillin/Streptomycin |
| SNU-349 | KCLB | 349 | | RPMI-1640, 10% (v/v) FCS, 1% (v/v) Penicillin/Streptomycin |
| SW-1710 | DSMZ | ACC 426 | Lot 3 | DMEM, 15% (v/v) FCS, 1% (v/v) Penicillin/Streptomycin |
| SW403 | DSMZ | ACC 294 | Lot 4 | DMEM, 10% (v/v) FCS, 1% (v/v) Penicillin/Streptomycin |
| SW48 | ATCC | CCL-231 | Lot 3247840 | DMEM, 10% (v/v) FCS, 1% (v/v) Penicillin/Streptomycin, 1 mM Sodium pyruvate |
| SW480 | ATCC | CCL-228 | Lot 4487738 | RPMI-1640, 10% (v/v) FCS, 1% (v/v) Penicillin/Streptomycin, 2 mM L-Glutamine, 1% (v/v) MEM non-essential amino acids, 1 mM Sodium pyruvate |
| SW620 | ATCC | CCL-227 | Lot 58483168 | RPMI-1640 10% (v/v) FCS, 1% (v/v) Penicillin/Streptomycin, 2 mM L-Glutamine, 1% (v/v) MEM NEAA |
| SW-780 | ATCC | CRL-2169 | Lot 61978343 | DMEM, 10% (v/v) FCS, 1% (v/v) Penicillin/Streptomycin |
| SW948 | ATCC | CCL-237 | Lot 62522213 | RPMI-1640, 10% (v/v) FCS, 1% (v/v) Penicillin/Streptomycin |
| T24 | DSMZ | ACC 376 | Lot 5 | DMEM, 10% (v/v) FCS, 1% (v/v) Penicillin/Streptomycin |
| T47D | ATCC | HTB-133 | | DMEM, 10% (v/v) FCS, 1% (v/v) Penicillin/Streptomycin, 1 mM Sodium pyruvate |
| T84 | ECACC | 88021101 | | Ham’s F12, 10% (v/v) FCS, 1% (v/v) Penicillin/Streptomycin, 2 mM Glutamine |
| TCC-SUP | DSMZ | ACC 377 | Lot 3 | DMEM, 15% (v/v) FCS, 1% (v/v) Penicillin/Streptomycin |
| ZR-75-1 | ATCC | CRL-1504 | | RPMI-1640, 10% (v/v) FCS, 1% (v/v) Penicillin/Streptomycin |
